# Mutations in Glycosyltransferases and Glycosidases: Implications for Associated Diseases

**DOI:** 10.3390/biom14040497

**Published:** 2024-04-19

**Authors:** Xiaotong Gu, Aaron S. Kovacs, Yoochan Myung, David B. Ascher

**Affiliations:** 1 School of Chemistry and Molecular Biosciences, University of Queensland, Brisbane, QLD 4000, Australia; xiaotong.gu@uq.edu.au (X.G.); a.kovacs@uq.edu.au (A.S.K.); y.myung@uq.edu.au (Y.M.); 2 Computational Biology and Clinical Informatics, Baker Heart and Diabetes Institute, Melbourne, VIC 3004, Australia

**Keywords:** glycosylation, glycosyltransferases (GTs), glycoside hydrolases (GHs), mutations, ClinVar, MTR score

## Abstract

Glycosylation, a crucial and the most common post-translational modification, coordinates a multitude of biological functions through the attachment of glycans to proteins and lipids. This process, predominantly governed by glycosyltransferases (GTs) and glycoside hydrolases (GHs), decides not only biomolecular functionality but also protein stability and solubility. Mutations in these enzymes have been implicated in a spectrum of diseases, prompting critical research into the structural and functional consequences of such genetic variations. This study compiles an extensive dataset from ClinVar and UniProt, providing a nuanced analysis of 2603 variants within 343 GT and GH genes. We conduct thorough MTR score analyses for the proteins with the most documented variants using MTR3D-AF2 via AlphaFold2 (AlphaFold v2.2.4) predicted protein structure, with the analyses indicating that pathogenic mutations frequently correlate with Beta Bridge secondary structures. Further, the calculation of the solvent accessibility score and variant visualisation show that pathogenic mutations exhibit reduced solvent accessibility, suggesting the mutated residues are likely buried and their localisation is within protein cores. We also find that pathogenic variants are often found proximal to active and binding sites, which may interfere with substrate interactions. We also incorporate computational predictions to assess the impact of these mutations on protein function, utilising tools such as mCSM to predict the destabilisation effect of variants. By identifying these critical regions that are prone to disease-associated mutations, our study opens avenues for designing small molecules or biologics that can modulate enzyme function or compensate for the loss of stability due to these mutations.

## 1. Introduction

Glycosylation is a post-translational modification that modulates the diverse functions of proteins and lipids in various cellular processes by attaching glycans. This modification not only influences the functionality and activity of these biomolecules but also significantly impacts their conformational stability and solubility. Glycosylation encompasses multiple types, each attaching sugar moieties to different amino acid residues or lipid molecules. These types include N-glycosylation and O-glycosylation, the most common forms involving the attachment of sugars to the nitrogen of asparagine residues, and the oxygen of serine/threonine, respectively. Other forms like C-glycosylation, glypiation and phosphoglycosylation attach sugars to unique amino acid residues or lipid structures, diversifying the functional impact of glycosylation [1]. Subtypes of O-linked glycosylation include O-N-acetylgalactosamine (O-GalNAc), primarily initiating mucin-type glycosylation; O-N-acetylglucosamine (O-GlcNAc), unique for its reversibility and regulation of cellular functions; O-Mannose (O-Man), significant in neural and muscle tissues; O-Galactose (O-Gal) and O-Fucose (O-Fuc), both crucial in protein interactions; and the less common O-Glucose (O-Glc), involved in specific developmental processes. While the majority of glycosylation events take place within the endoplasmic reticulum (ER) and Golgi apparatus, primarily mediated by enzymatic reactions involving glycosyltransferases and glycoside hydrolases, the lysosome’s role in glycan breakdown is also essential. Lysosomal glycoside hydrolases are essential, breaking down complex sugars into monosaccharides that the cell can then repurpose, balancing the synthetic activities of glycosyltransferases [2]. This dynamic between synthesis and degradation sustains the glycan lifecycle, influencing the myriad biological functions these enzymes facilitate. Due to the myriad of potential glycan structures that can be attached to proteins, a wide array of enzymes is engaged in the synthesis of diverse glycoconjugates.

Glycosyltransferases (GTs) play a pivotal role in the formation of glycosidic bonds, enabling the attachment of sugar moieties to proteins, lipids or other sugars [3]. These enzymes are classified into distinct categories, GT-A and GT-B, based on the specific glycosidic bonds they form. GT-A enzymes exhibit a defined structure characterised by a metal-coordinating DxD motif, while GT-B enzymes possess a more flexible structure [4], allowing them to accommodate various sugar substrates. Given the multifaceted roles of GTs in cellular and metabolic processes, including protein folding, stability, cell–cell adhesion, signalling and adaptive immune responses, malfunctions or mutations in GTs have been linked to a spectrum of diseases [5,6].

Glycoside hydrolases (GHs), also known as glycosidases, are enzymes that catalyse the hydrolytic cleavage of glycosidic bonds, facilitating the degradation of carbohydrates and glyco-molecules [7]. GHs can be categorised based on their mode of cleavage, whether from internal or terminal positions within glycosidic bonds. Furthermore, GHs can be classified according to their specificity for particular sugar moieties [8], such as glucose, galactose or mannose, through the action of glucosidases, galactosidases and mannosidases, respectively. GHs can also be differentiated by their mechanism of action into “inverting” and “retaining” enzymes [9], which refer to their impact on the stereochemistry of the carbon atom at the isometric centre of the sugar molecule. These distinctive features of GHs have led to their widespread application in catalysing specific chemical reactions, enhancing our comprehension of the complexity and specificity of biochemical processes in various domains.

Glycosyltransferases are involved in the biosynthesis of glycoconjugates and are crucial for the addition of sugar moieties from activated donor molecules to specific acceptor molecules, forming glycosidic bonds, in processes such as N-linked glycosylation, O-GlcNAcylation, glycolipid biosynthesis and beyond. Glycosidases, on the other hand, play a key role in cellular degradation pathways by hydrolysing glycosidic bonds in a range of biomolecules. Glycosidases are instrumental not only in lysosomal degradation but also in the ER-associated degradation process, which is essential for maintaining protein quality control [10]. Furthermore, glycosidases are involved in the precise processing of glycans, which is vital for proper cellular function and homeostasis.

Variants in glycosyltransferases can manifest as subtle alterations in enzyme activity or substrate specificity, or they can lead to severe disruptions associated with genetic disorders. Notable examples include allergic reactions triggered by GT-As, blood group determinants influencing transfusion compatibility [11], and even responses to specific medications. On the other hand, GT-B variants have been associated with conditions such as muscle and brain abnormalities [12], drug resistance [13] and congenital disorders of glycosylation [14]. Compared to GTs, variants in GHs predominantly result in diseases associated with the accumulation of undegraded or incompletely degraded glycan substrates. Notable examples include Gaucher’s disease, Fabry disease and Pompe disease, which exhibit skeletal abnormalities, neurological disorders and muscle weakness [15]. Additionally, lactose intolerance, characterised by gastrointestinal symptoms due to the inability to break down lactose, is a common consequence of GH deficiencies [16].

Consequently, comprehending the diverse effects of these variants is paramount, as it holds significant implications for both fundamental research and clinical applications. Such insights shed light on the underlying mechanisms of glycosylation-related diseases and open avenues for the identification of potential therapeutic targets.

While extensive research has explored the role of glycoproteins in health and disease, there remains a notable gap in our understanding regarding the effects of missense mutations in glycosylating proteins, their sequential and structural aspects and their implications in disease pathogenesis. In this comprehensive review, we aim to delve into the intricate world of glycosyltransferases and glycoside hydrolases.

## 2. Variants of Human Glycosyltransferases and Glycoside Hydrolases in ClinVar

As of December 2023, out of the 4,779,468 entries in ClinVar, 128,468 are categorised as “single nucleotide variants”. These entries exclude “Ter” (termination) variants and lack an assertion of “Review Status”. Information on variants of human glycosyltransferases (GTs) and glycoside hydrolases (GHs) was compiled from 343 Gene IDs sourced from UniProt, classified under the enzyme commission numbers E.C 2.4.-.- and E.C 3.2.-.- for GTs and GHs, respectively. Overall, the ratio of benign to pathogenic variants reported in ClinVar is nearly 2:1, with 87,254 benign and 41,214 pathogenic variants. Upon applying filters to the ClinVar entries, focusing on GT and GH genes, we identified 2603 entries that could be mapped to UniProt IDs. Among these, 1307 were classified as benign variants, while 1296 were identified as pathogenic variants.

**Figure 1:**
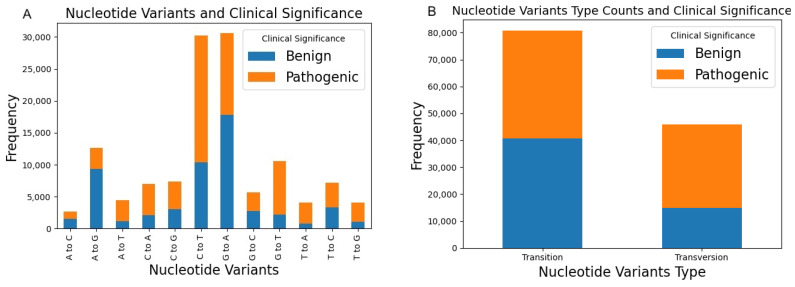
Clinical significance of nucleotide variants in GT/GH genes. (**A**) The frequency of specific nucleotide substitutions. (**B**) The total counts of transition and transversion types. The *x*-axis specifies the variant types or categories, while the *y*-axis quantifies their occurrence.

At the nucleotide level, we examined the frequency of variants for the adenine (A), cytosine (C), guanine (G) and thymine (T) molecules. Higher GC content, denoting a greater proportion of G and C nucleotides, typically correlates with increased stability of DNA molecules due to the stronger hydrogen bonds between G and C nucleotides compared to A and T nucleotides. Consequently, regions with higher GC content tend to be more resistant to mutation-induced changes, maintaining genetic information across generations [17]. Following this theory, this study assesses a basic evaluation of nucleotide variants in proteins to discern any potential association between the prevalence of GC content variants and the ClinVar clinical significance (see Figure 1). The summarisation of the mutation type comparison between transition and transversion aligned with the recognised transition: transversion bias, where transition mutations manifest considerably more frequently than anticipated under uniformity [18]. Further, as depicted in Figure 1, we categorised the nucleotide variants into transition and transversion types and correlated these with their clinical significance as determined by ClinVar. Figure 1A illustrates the frequencies of each nucleotide variant, providing insight into the overall mutation landscape, while Figure 1B specifically highlights the clinical relevance of these mutations, distinguishing between benign and pathogenic outcomes. Notably, pathogenic mutations appear more frequently in transition types, suggesting a higher risk of pathogenicity in these mutations.

**Figure 2:**
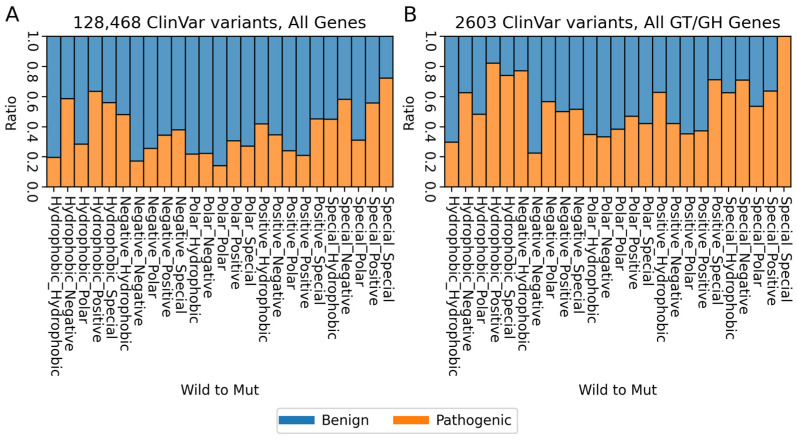
Pharmacophore change ratio between benign and pathogenic variants. Mapping of pharmacophore ratios is depicted for (**A**) 128,468 ClinVar variant entires spanning all genes and (**B**) 2603 ClinVar variants associated with GT/GH genes. The pharmacophore categories include“Positive” (Arg, His, Lys), “Negative” (Asp, Glu), “Polar” (Ser, Thr, Asn, Gln), “Special” (Cys, Gly, Pro) and “Hydrophobic” (Ala, Val, Ile, Leu, Met, Phe, Tyr, Trp).

At the sequence level, a pharmacophore analysis of 128,468 variants indicates that substitutions from “standard” amino acids to “special” ones, such as proline, glycine and cysteine, are highly pathogenic across each category. These “special” amino acids are characterised by their substantial impact on protein backbone structure. Proline, with its cyclic structure, introduces rigidity, often leading to disruptions in secondary structures. Glycine, being exceptionally small, provides flexibility and is typically found in the tight turns of proteins. Cysteine’s unique ability to form disulfide bonds is essential for the stability and conservation of tertiary and quaternary protein structures. This is particularly evident in substitutions from “Special” to “Special” amino acids (see Figure 2A). Notably, changes from “Hydrophobic” to “Positive” amino acids and vice versa exhibit a significantly high pathogenicity ratio in GT/GH genes (Figure 2B). Furthermore, 11 instances of “Special to Special” substitutions are identified as 100% pathogenic, comprising 6 “Cysteine to Glycine” and 5 “Glycine to Cysteine” changes. This aligns with existing research [19] highlighting the critical role of cysteine in protein stability and conservation. Glycine, often found at the turns of alpha-helices, might adversely affect protein structural integrity.

**Figure 3:**
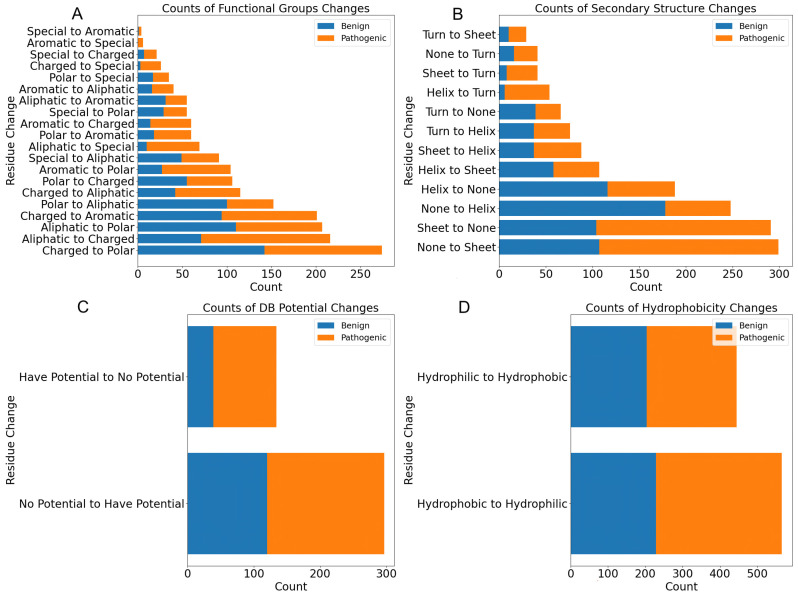
Distribution of amino acid changes in benign and pathogenic variants in GT/GH proteins. (**A**) Functional group alterations, counting the shifts in amino acid properties. (**B**) Structural transitions, illustrating changes in amino acids affecting protein secondary structure. (**C**) Disulfide bond potential, comparing the potential for forming or disrupting disulfide bonds. (**D**) Hydrophobicity shifts, summarising the transition between hydrophilic and hydrophobic amino acids. The *x*-axis quantifies the number of observed substitutions for each category while the *y*-axis lists changes categories.

Additionally, our analysis reveals that the GT/GH gene variants shifting from “Negative” to “non-Negative” amino acids are more prevalent compared to the overall rate of “Negative” variants. This is particularly significant considering the role of negative amino acids like Glutamate (Glu) and Aspartate (Asp) in catalytic mechanisms. Such alterations could substantially impact the stability and functionality of the enzymes. This finding highlights the critical nature of these specific amino acids in maintaining enzyme integrity and suggests that even minor shifts in their composition can lead to significant functional consequences. Therefore, these findings underscore that structural instability, driven by such amino acid substitutions, can contribute to the pathogenicity of GT/GH genes. Also, in Figure 3, we illustrate the comparative alteration in pharmacophore ratios between benign and pathogenic variants, outlining the mapping of distinct pharmacophore categories: “Positive” (Arg, His, Lys), “Negative” (Asp, Glu), “Polar” (Ser, Thr, Asn, Gln), “Special” (Cys, Gly, Pro) and “Hydrophobic” (Ala, Val, Ile, Leu, Met, Phe, Tyr, Trp) for both all gene analysis and the selected GT/GH gene variants.

Out of the 2603 GT/GH variants catalogued in ClinVar, Table 1 highlights the top 20 genes with the highest variant counts (combining benign and pathogenic variants). These genes represent promising candidates for further in-depth structural studies, given their abundant variant data and associated structural information. The enzymes listed, considering the balance between synthesis and degradation, were also curated based on their involvement in either the lysosomal breakdown of glycoconjugates or glycoprotein biosynthesis. The abundant variant data for these enzymes facilitate the understanding of significant features that are consequential to enzyme function and, by extension, to cellular processes and pathophysiology. This selection offers valuable insights into the effects of mutations from a structural perspective.

As shown in Figure 3, we also present a comprehensive series of bar charts that collectively offer a multi-faceted view of amino acid substitution effects within GT/GH gene structures. From Figure 3A, we noticed that the most frequent transitions observed in pathogenic mutations involve changes from “Aliphatic to Charged” followed by “Aliphatic to Polar” and “Charged to Polar”. This suggests that pathogenic variants often result in the introduction of charged or polar residues in place of nonpolar, aliphatic ones, potentially altering the electrostatic landscape and interaction capabilities of the protein. As shown in Figure 3B, secondary structure changes are dominated by transitions from turn to none, which could indicate a loss of structural definition, while hydrophobicity changes display a balanced occurrence of shifts from hydrophilic to hydrophobic residues and vice versa, reflecting the amphipathic nature of protein interaction surfaces. Figure 3C illustrates changes in the potential for disulfide bond (DB) formation, where all amino acids are initially classified as lacking DB potential, while cysteine (Cys) and histidine (His) residues are specifically labeled as having “DB Potential”. This result highlights a considerable number of substitutions that increase the likelihood of disulfide bond formation, aligning with the possibility of correlating with an increased frequency of pathogenic missense variations. The shift towards hydrophobicity in pathogenic variants (Figure 3D) indicates an increased tendency for these mutations to be buried within the protein core, potentially disrupting internal interactions and the protein’s hydrophobic-hydrophilic balance that links to pathogenic missense mutations.

**Figure 4:**
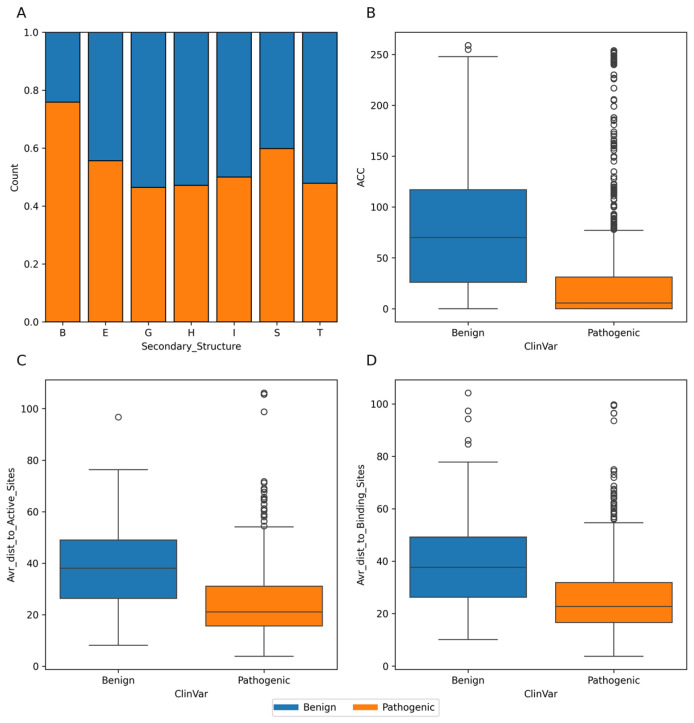
Structural analysis of GT/GH proteins. (**A**) Distribution of variants across different secondary structures. (**B**) Solvent-accessible area calculations for variants using the DSSP toolkit (MKDSSP V3.0.0) on wild-type AlphaFold2 structures. DSSP classifies protein secondary structure into seven main elements: Alpha Helix {H}s, Beta Bridge {B}, Extended Strand {E}, 3/10 Helix {G}, Pi Helix {I}, Turn {T} and Bend {S}. Each element corresponds to specific structural patterns within the protein’s atomic coordinates. (**C**) Average distance of variants to the nearest active site, crucial for the catalytic activity of enzymes. (**D**) Average distance of variants to the nearest binding site, indicating the potential impact on molecular interactions. These distances were calculated for the top 20 genes using UniProt data.

In our analysis of 2603 ClinVar variants, a noteworthy pattern emerges: pathogenic mutations tend to be more frequently associated with the Beta Bridge secondary structure element (Figure 4A). This finding aligns harmoniously with the critical role of beta sheets in maintaining the functional and structural integrity of both GT-A (DXD motif) and GT-B (Rossmann-like domains). Furthermore, our investigation reveals that benign variants generally exhibit higher solvent accessibility (ACC) compared to their pathogenic counterparts (Figure 4B). This observation suggests that pathogenic mutations are predominantly located within the inner regions of proteins, a phenomenon with potentially profound implications for protein stability. Examining the average distances to active (Figure 4C) and binding sites (Figure 4D) on the top 20 GT/GH genes provides insight into the mutation’s functional implications. Pathogenic variants are often closer to active sites, possibly affecting catalytic activity, and to binding sites, potentially disrupting essential molecular interactions. This analysis highlights how the spatial positioning of mutations in relation to these sites can have profound effects on protein functionality.

## 3. Comprehensive Structural Analysis of Glycosyltransferases and Glycoside Hydrolases (GT/GHs)

In this section, we integrate the MTR3D-AF2 analytical platform with the predictive power of AlphaFold2 to determine missense variation scores (MTR) and interpret and visualise the structural repercussions of genetic variants within the genes. MTR3D-AF2 is a recently launched server that enables the visualisation of MTR and MTR3D [20] scores across more than 17,000 human genes. MTR3D-AF2 assesses missense intolerance by quantifying the degree to which variation is depleted across the human population. In cases where the MTR3D-AF2 scores are not available, MTR3D scores (https://biosig.lab.uq.edu.au/mtr3d/) are used instead, as accessed on 1 December 2023. All MTR3D-AF2 scores in this analysis were calculated using 8Å windows and can be downloaded and visualised via the MTR3D-AF2 webserver (https://biosig.lab.uq.edu.au/mtr3daf2/, accessed on 1 December 2023).

We also analysed a series of mutations within selected enzymes to assess their potential impact on protein function and structure. The accessibility of the variant residue within the protein structure is calculated, measuring the surface area accessible to solvent molecules. Moreover, we calculated the maximum, minimum and average distance as well as standard deviation from the mutations to denoted active sites from UniProt records within the protein. In a similar vein, we appraised the variants’ maximum, minimum and average distance, as well as standard deviation from denoted binding sites. To assess the effects of the mutations on protein stability, we utilised the mCSM [21], which employs graph-based signatures to gauge the impact of point mutations on protein stability, with negative values intimating a destabilising effect. We also employed normal mode analysis (NMA) to examine the dynamics in enzymes. NMA is a method used to evaluate protein atom movements by assessing vibrational modes [22]. We measured deformation and fluctuation scores for each residue to estimate the protein’s local and global flexibility. The lower deformation energy suggests increased flexibility in the protein structure, while higher values indicate rigidity and possible functional constraints. In addition, we employ Rate4Site (r4s) [23] to calculate the conservation scores of amino acids within proteins, which enables us to identify regions of high evolutionary conservation that are likely critical for the potential impact of mutations on protein activity. The medians and Mann–Whitney test results for the studied features of each selected protein are provided in Table S1 of the Supplementary Material.

The figures in this section for each enzyme offer a comprehensive view, starting with a three-dimensional representation of the enzymes, where pathogenic variants are highlighted in magenta and benign variants in blue. The secondary structure is classified using the DSSP algorithm, and variants are categorised accordingly, providing insight into the structural contexts prone to pathogenic versus benign variants. Box plots elucidate the solvent-accessible surface areas of these variants, shedding light on how each variant might interact with the surrounding environment—a factor that can significantly impact molecular interactions and enzyme activity. The MTR3D-AF2-generated graphs offer a granular view of the impact of variants along the protein sequence. Here, the *y*-axis measures the predicted impact on protein stability, and the *x*-axis lists the amino acid positions. Benchmark lines in green, yellow and red indicate neutral, moderate and high destabilising effects, respectively, with variants above the red line suggesting significant structural perturbations.

### 3.1. Alpha-Galactosidase A, GLA

Alpha-Galactosidase A is an enzyme responsible for catalysing the hydrolysis of α-D-galactose residues, both terminal and non-reducing, within the lysosome. This lysosomal enzyme possesses definitive active sites at amino acid residues 170 as a nucleophile and 231 as a proton donor. The substrate’s binding site is located across amino acids 203 to 207, indicating an important region for substrate recognition and binding, upon which GLA acts. GLA is localised within the lysosome, an organelle encased by a membrane that contains hydrolytic enzymes responsible for the breakdown of various biomolecules. In terms of structure, GLA operates as a homodimer, comprising two identical subunits of a homodimer combined to form the active enzyme [24].

**Figure 5:**
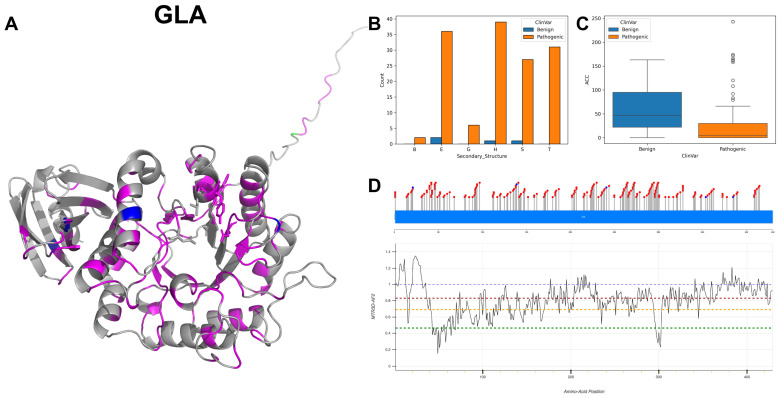
Structural analysis of the GLA protein. (**A**) Benign variants are depicted in blue, while pathogenic variants are highlighted in magenta. Active and binding sites are illustrated in stick representation. (**B**) Variant locations within the secondary structure are indicated. (**C**) Solvent accessibility distribution is determined using the DSSP toolkit. (**D**) Assessment of ClinVar missense variants is presented in a lollipop plot, with benign variants in blue and pathogenic variants in red. The impact of these variants’ tolerance on the AlphaFold2 structure is evaluated through MTR3D-AF2. Different colored dotted lines indicate gene-specific MTR percentiles, with green, yellow, red, and blue denoting the 5th, 25th, 50th, and neutrality (MTR = 1) percentiles, respectively.

Among the GT/GH gene family, GLA exhibits the highest number of reported missense variants, as indicated in Table 1, with a total of 207 variants documented in ClinVar. Notably, when examining the location of these pathogenic variants (depicted in magenta in Figure 5A), they tend to cluster in proximity to active and binding sites, as illustrated in the stick representation within the same figure. GLA is associated with a spectrum of clinical conditions, prominently including Fabry disease and various cardiovascular phenotypes. Analysis of mutations in GLA reveals a distribution wherein transition mutations predominate over transversions, with transitions comprising 127 occurrences compared to 80 transversions. Among nucleotide variants, the most frequent are G to A alterations with 46 occurrences. The 3D and secondary structure (Figure 5A,B) calculation shows a predominance of alpha-helices and beta-strands. The ACC (Figure 5C) is notably lower than the ACC of benign variants, suggesting most documented pathogenic variants are likely buried. As shown in Figure 5D, the segment of GLA characterised most consistently by the lowest MTR3D-AF2 scores, specifically residues 45 to 69, is one of the regions most enriched with pathogenic variants as compared to benign variants. Investigating the clinical significance associated with GLA mutations, a total of 202 instances are marked as pathogenic. Conversely, only a marginal count of five for benign is documented. The phenotypic manifestations linked to GLA mutations portray a predominant occurrence of angiokeratoma corporis diffusum with 168 documented instances [25]. Variants of GLA have been implicated in Fabry disease [26], rare X-linked sphingolipidosis-related glycosphingolipids in plasma and cellular lysosomes across the body.

In the comparison of molecular consequences between GLA pathogenic mutations (*n* = 202) and benign variants (*n* = 5), it is observed that pathogenic variants are typically more buried within the protein structure, with a median ACC of 1.0 Å versus 22.0 Å for benign variants. Additionally, pathogenic variants are, on average, slightly closer to both active sites (18.42 Å vs. 26.77 Å) and binding sites (20.64 Å vs. 27.98 Å). Regions harbouring both pathogenic and benign variants show low fluctuation energies, with median NMA values of 0.06 Å^2^ for pathogenic and 0.09 Å^2^ for benign variants. It is noteworthy that pathogenic variants within these regions are predominantly located in areas characterised by comparatively higher rigidity (median NMA 0.23 Å^2^) as opposed to benign variants (median NMA 0.48 Å^2^), indicating possibly deformation energy and reduced conformational flexibility. Pathogenic variants are also found to be more evolutionarily conserved (r4s *p*-value = 4.16 × 10^−4^), whereas the changes in stability, as predicted by mCSM scores, are not statistically significant (mCSM *p*-value = 0.69) when compared to benign variants.

### 3.2. Lysosomal Alpha-Glucosidase, GAA

GAA encodes the enzyme acid alpha-glucosidase, which is key to the catabolism of glycogen to glucose within lysosomes. GAA’s activity is facilitated by two active sites at amino acid positions 518, functioning as a nucleophile, and 521 [27]. GAA also exhibits substrate-binding sites at amino acid positions 404, 600, 616 and 674, which is necessary for the enzymatic action. GAA is localised within the lysosomal compartment [28], both within the lumen and integrated into the lysosomal membrane.

**Figure 6:**
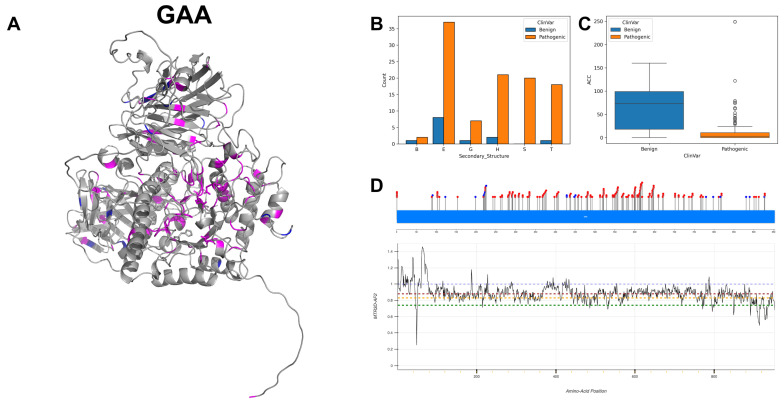
Structural analysis of the GAA protein. (**A**) Localisation of benign variants in blue and pathogenic variants in magenta. (**B**) Annotated secondary structure variant mapping. (**C**) ACC distribution. (**D**) Assessment of ClinVar missense variants is presented in a lollipop plot, with benign variants in blue and pathogenic variants in red. The impact of these variants’ tolerance is evaluated through MTR3D-AF2, with green, yellow, red, and blue denoting the 5th, 25th, 50th, and neutrality (MTR = 1) percentiles, respectively.

The mutational landscape of GAA reveals a notable prevalence of mutations, with nucleotide transitions of G to A leading in frequency, accounting for 49 occurrences, followed by C to T mutations with 24 instances. As shown in Figure 6A,B, the variants, both pathogenic and benign, mainly occur in extended strands. The ACC (Figure 6C) range of pathogenic variants is significantly lower than benign scores, indicating that most documented pathogenic variants are possibly buried. The majority of residues across GAA (Figure 6D) are associated with MTR3D-AF2 scores ranging between 1 and 0.8, suggesting that only a small amount of missense intolerance is found throughout the gene. The distribution between benign and pathogenic classifications emphasises a predominance of pathogenic mutations, with a count of 150 instances compared to 17 benign occurrences. Clinical phenotypes attributed to mutations in the GAA gene are primarily represented by glycogen storage disease, type II [29]. A discernible representation of cardiovascular phenotypes is also observed in the clinical spectrum [30] associated with GAA gene mutations.

In comparing the molecular outcomes of GAA pathogenic mutations (*n* = 150) with benign variants (*n* = 17), we note that pathogenic variants are more often found closer to the protein core (*p*-value = 2.36 × 10^−5^), with an ACC of 0 Å against 26.5 Å for benign variants. Pathogenic variants also tend to be nearer to active sites (*p*-value = 3.26 × 10^−5^, median distance of 19.07 Å vs. 37.50 Å for benign variants) and binding sites (*p*-value = 2 × 10^−4^, median distance of 22.27 Å vs. 37.55 Å for benign variants). In areas containing both variant types, pathogenic variants tend to appear at locations with lower fluctuation energies (median NMA of 0.020 Å^2^) compared to benign variants (median NMA of 0.07 Å^2^), although this result is not statistically significant (*p*-value = 4.07 × 10^−3^). Interestingly, pathogenic variants tend to be located in areas with greater rigidity (median NMA of 0.129 Å^2^ vs. 0.149 Å^2^ for benign variants), yet this observation does not reach statistical significance (*p*-value = 0.49). In addition, pathogenic variants are more evolutionarily conserved (*p*-value = 2.73 × 10^−6^), as shown by a significantly lower median r4s score of 0.52 compared to 2.33 for benign variants. Stability changes predicted by mCSM scores do not differ significantly between pathogenic and benign variants (*p*-value = 4.44 × 10^−4^), despite a trend towards lower stability in pathogenic variants (median mCSM of −1.17 vs. −0.40 for benign variants).

### 3.3. Beta-Galactosidase, GLB1

GLB1 codes for the enzyme beta-galactosidase, and its activity hinges on two active sites located at amino acids 188 and 268. At spot 188, the enzyme functions as a proton donor, aiding the breakdown of substrates through proton donation [31], while the other active site at 268 acts as a nucleophile, crucial for providing electrons for bond formation. GLB1 also boasts several essential substrate-binding sites at residues 83, 129, 187 and 333 [32]. GLB1’s subunit structure is a homodimer, comprising two identical subunits, and might also form larger multimeric structures [33]. The subcellular localisation of GLB1 differs between its isoforms. Isoform 1 is mainly found in the lysosome, crucial for its role in breaking down glycosphingolipids [34]. On the flip side, Isoform 2 is spotted in the cytoplasm, particularly near the nucleus, and is notably missing from lysosomes.

**Figure 7:**
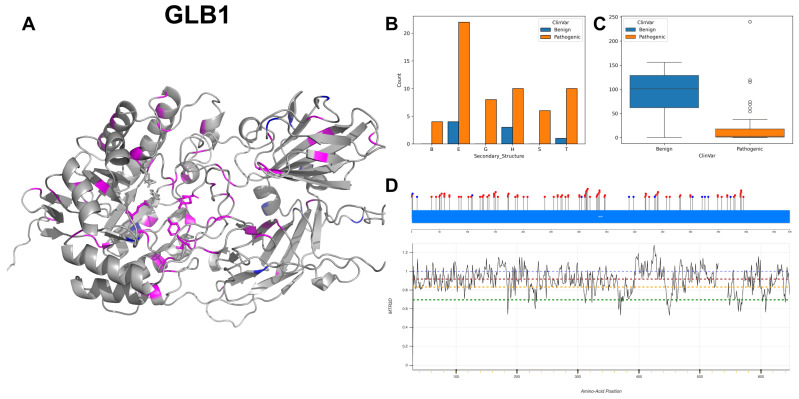
Structural analysis of the GLB1 protein. (**A**) Localisation of benign variants in blue and pathogenic variants in magenta. (**B**) Annotated secondary structure variant mapping. (**C**) ACC distribution. (**D**) Assessment of ClinVar missense variants is presented in a lollipop plot, with benign variants in blue and pathogenic variants in red. The impact of these variants’ tolerance is evaluated through MTR3D-AF2, with green, yellow, red, and blue denoting the 5th, 25th, 50th, and neutrality (MTR = 1) percentiles, respectively.

G to A and C to T transitions of GLB1 lead nucleotide variant count with 22 and 18 instances, respectively. The secondary structure components of GLB1 (Figure 7B) show a predominant abundance of alpha-helices and beta-strands, coinciding with the 3D structure (Figure 7A). The ACC range of pathogenic variants (Figure 7C) shows a considerably constricted interquartile range and lower media value, indicating more buried locations. The residues across GLB1 are associated with MTR3D scores ranging between 1 and 0.75 (Figure 7D) suggesting that a consistent and small amount of missense intolerance is found throughout the gene. The three benign residues falling within the range of 400 to 440, with MTR3D scores ranging from 1.1 to 1.25, are clearly indicative of a heightened tolerance to missense variations. Clinical implications linked to mutations encompass a spectrum of disorders, mainly for GM1 gangliosidosis [31], with a predominant count of pathogenic variants numbering 85 instances, in contrast to 12 benign variants out of a total of 97 documented occurrences.

In examining the molecular characteristics of GLB1 pathogenic mutations (*n* = 85) against benign variants (*n* = 12), we find that pathogenic mutations are significantly closer to the protein core (*p*-value = 1.41 × 10^−2^), with a median ACC of 2.5 Å compared to 67 Å for benign variants. Additionally, pathogenic variants have a closer median distance to active sites (22.02 Å vs. 41.87 Å for benign variants) and biding sites (22.87 Å vs. 36.38 Å for benign variants), although these differences do not reach statistical significance (*p*-value = 9.69 × 10^−2^ and *p*-value = 0.123, respectively). In the analysis of structural dynamics, pathogenic variants tend to appear at regions that are more rigid (NMA of 0.081 Å^2^ vs. 0.096 Å^2^ for benign variants), while this observed increase in deformation does not present a significant difference (*p*-value = 0.747). Furthermore, regions that house pathogenic variants also display a tendency towards reduced flexibility (NMA of 0.057 Å^2^ vs. 0.218 Å^2^ for benign variants), yet this fluctuation difference is not statistically significant (*p*-value = 6.42 × 10^−2^). Pathogenic variants are more evolutionarily conserved and less tolerant to change (r4s *p*-value = 4.50 × 10^−3^, median of 0.695 vs. 2.339 for benign variants). In terms of stability, pathogenic variants tend to be less stable (mCSM *p*-value = 6.49 × 10^−2^, −1.367 vs. −0.531 for benign variants).

### 3.4. Galactocerebrosidase, GALC

GALC encodes the enzyme galactosylceramidase, which is integral to the lysosomal metabolism of galactolipids. GALC is characterised by two active sites at amino acid residues 198 identified as a proton donor/acceptor and 274 acting as a nucleophile. GALC also possesses substrate-binding sites at positions 109, 151, 197 and 396 that interact with the substrate [35]. GALC is localised within the lysosome, which is vital for the breakdown of galactolipids.

**Figure 8:**
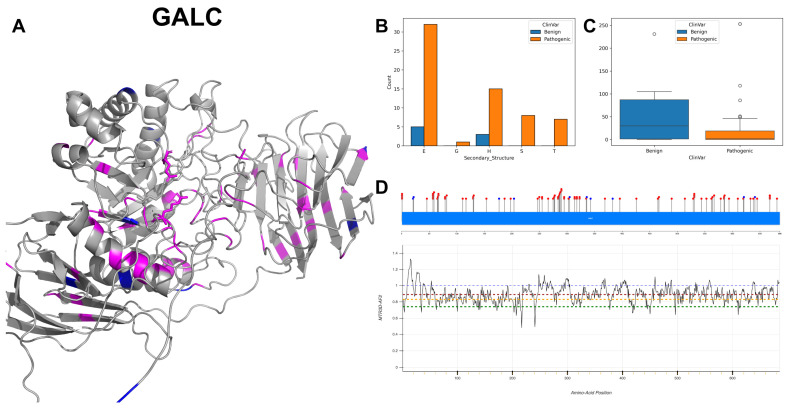
Structural analysis of the GALC protein. (**A**) Localisation of benign variants in blue and pathogenic variants in magenta. (**B**) Annotated secondary structure variant mapping. (**C**) ACC distribution. (**D**) Assessment of ClinVar missense variants is presented in a lollipop plot, with benign variants in blue and pathogenic variants in red. The impact of these variants’ tolerance is evaluated through MTR3D-AF2, with green, yellow, red, and blue denoting the 5th, 25th, 50th, and neutrality (MTR = 1) percentiles, respectively.

GALC exhibits a varied mutational spectrum, with the G to A transitions dominating the DNA coding variant landscape with 22 instances, followed by A to G and G to T alterations, both documented in 11 instances. The structural analysis of GALC (Figure 8A,B) indicates a significant prevalence of beta-strands, and the pathogenic variants exhibit not only a lower ACC (Figure 8C) but also a narrowed interquartile range, suggesting a more constrained structural conformation compared to benign variants. The majority of residues across GALC are associated with MTR3D-AF2 scores ranging between 1 and 0.75 (Figure 8D), suggesting that a consistent and small amount of missense intolerance is found throughout the gene. GALC’s mutations carry clinical implications marked by a prevalence of pathogenic variants. Clinical associations of mutations in GALC correlate with Galactosylceramide beta-galactosidase deficiency [36], displaying a significant count of 82 instances.

Upon reviewing the molecular characteristics of GALC pathogenic mutations (*n* = 80) against benign variants (*n* = 10), we note a non-significant difference in the proximity to the protein core with a median ACC of 0 Å for pathogenic and 7.5 Å for benign variants (*p*-value = 0.19). The pathogenic variants are significantly closer to both active sites (20.34 Å vs. 26.27 Å for benign variants, *p*-value = 3.28 × 10^−2^) and binding sites (20.19 Å vs. 28.05 Å for benign variants, *p*-value = 9.2 × 10^−3^). In the analysis of structural dynamics, pathogenic variants are found in the regions that are possibly more rigid (deformation *p*-value = 0.616 with median NMA of 0.003 Å^2^ versus 0.005 Å^2^ for benign variants) and less flexible (fluctuation *p*-value = 0.038 with median NMA of 4 × 10^−4^ Å^2^ vs. 7 × 10^−4^ Å^2^ for benign variants). Evolutionary conservation analysis indicates that pathogenic variants tend to be more conserved and less tolerant to alterations (r4s *p*-value = 1.71 × 10^−3^, median of 0.796 for pathogenic vs. 1.420 for benign variants). Finally, stability assessment through mCSM does not reveal a significant difference between pathogenic and benign variants (*p*-value = 0.985 with a median of −1.036 vs. −0.967, respectively), suggesting a comparable level of stability between the two.

### 3.5. Alpha-N-Acetylgucosaminidase, NAGLU

NAGLU encodes the enzyme alpha-N-acetylglucosaminidase, which is critical in the degradation of glycosaminoglycans in the lysosome [37]. NAGLU functions both as a monomer and a homodimer, indicating that it can operate as a single polypeptide or as a complex of two identical subunits. The subcellular location of this enzyme within the lysosome is crucial for the degradation of glycosaminoglycans, preventing their accumulation which could lead to cellular dysfunction.

**Figure 9:**
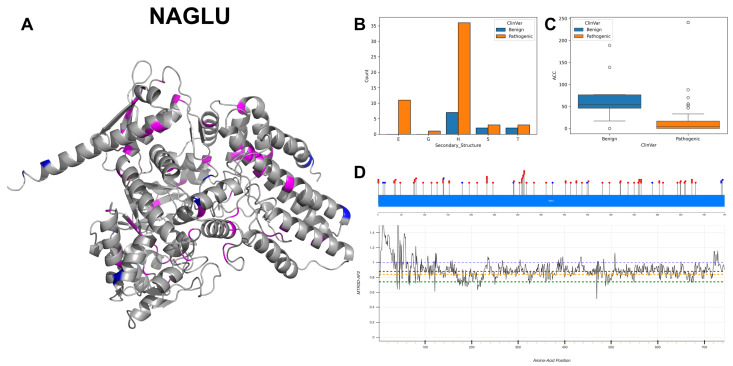
Structural analysis of the NAGLU protein. (**A**) Localisation of benign variants in blue and pathogenic variants in magenta. (**B**) Annotated secondary structure variant mapping. (**C**) ACC distribution. (**D**) Assessment of ClinVar missense variants is presented in a lollipop plot, with benign variants in blue and pathogenic variants in red. The impact of these variants’ tolerance is evaluated through MTR3D-AF2, with green, yellow, red, and blue denoting the 5th, 25th, 50th, and neutrality (MTR = 1) percentiles, respectively.

Variations of NAGLU exhibit diverse mutations, with a prominent occurrence of C to T transition at 16 occurrences, as well as G to A and T to C mutations at 15 and 12 counts, respectively. The 3D and secondary structure composition of NAGLU (Figure 9A,B) shows a significant prevalence of alpha-helices with most benign variants. The ACC values of pathogenic variants (Figure 9C) display a reduced median score compared with benign variants, indicating lower accessibility. While the majority of the residues across the gene are associated with scores ranging from 1 to 0.75 (Figure 9D), there is a segment at the beginning of the gene, specifically from residues 0 to 35, where scores are >1. In this region, there is a cluster of four benign variants and one pathogenic variant. At the end of the gene, from residues 720 to 744, all are associated with scores > 0.9. In this region, a cluster of three benign variants can be found. Among these, 72 variants are classified as pathogenic, while 11 instances are considered benign, delineating a significant predominance of pathogenic alterations. NAGLU is documented associated with two main conditions: Mucopolysaccharidosis, MPS-III-B [38], a lysosomal storage disorder with severe neurological symptoms, and Charcot–Marie–Tooth disease axonal type 2V, a peripheral neuropathy.

Upon investigating the molecular features of NAGLU pathogenic mutations (*n* = 72) in comparison to benign variants (*n* = 11), our findings reveal that pathogenic mutations are significantly nearer to the protein’s core (*p*-value = 2.8 × 10^−3^), with a median ACC of 1 Å for pathogenic mutations and 59.5 Å for benign variants. In the context of structural dynamics, pathogenic mutations are found in regions exhibiting increased rigidity (NMA of 0.072 Å^2^ vs. 0.264 Å^2^ for benign variants), though this increase in rigidity does not reach statistical significance (*p*-value = 0.034). Conversely, pathogenic variants reside in areas of decreased flexibility (NMA of 0.024 Å^2^ vs. 0.060 Å^2^ for benign variants); this difference, however, is not statistically significant (*p*-value = 0.167). Notably, pathogenic variants are characterised by higher evolutionary conservation and lower tolerance to change (r4s *p*-value = 0.366), with a median of 0.765 vs. 1.203 for benign variants. Regarding stability, pathogenic variants tend to be less stable (mCSM *p*-value = 8.4 × 10^−4^), showcasing a median of −1.377 vs. −0.196 for benign variants.

### 3.6. Alpha-L-Iduronidase, IDUA

IDUA encodes the enzyme alpha-L-iduronidase, critical for the lysosomal degradation of glycosaminoglycans, specifically dermatan sulfate and heparan sulfate. IDUA’s functionality is facilitated by active sites at amino acid positions 182 and 299. At position 182, it acts as a proton donor, a role vital for the hydrolysis reaction, while the active site at position 299 serves as a nucleophile, engaging in the chemical reaction necessary to break the glycosidic bonds within the substrate [39]. IDUA has a complex substrate-binding profile, engaging with alpha-D-mannopyranose and alpha-L-iduronate at multiple sites, notably residues 54, 56, 58, 91, 181, 182, 264, 299, 305, 306, 349, 363, 488 and 492 [40]. IDUA is described as functioning in a monomeric state within the lysosome, and its lysosomal location is critical for its role in the catabolism of glycosaminoglycans, preventing their pathological accumulation.

**Figure 10:**
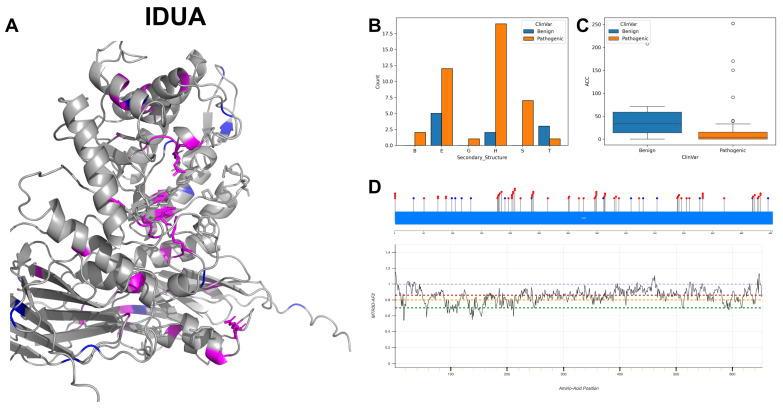
Structural analysis of the IDUA protein. (**A**) Localisation of benign variants in blue and pathogenic variants in magenta. (**B**) Annotated secondary structure variant mapping. (**C**) ACC distribution. (**D**) Assessment of ClinVar missense variants is presented in a lollipop plot, with benign variants in blue and pathogenic variants in red. The impact of these variants’ tolerance is evaluated through MTR3D-AF2, with green, yellow, red, and blue denoting the 5th, 25th, 50th, and neutrality (MTR = 1) percentiles, respectively.

The investigation into IDUA gene mutations reveals a relatively balanced distribution between transition and transversion variant types, with 39 transitions and 36 transversions observed. Notably, G to A alteration is the most frequent, appearing 19 times, followed by C to G and G to C mutations, each occurring 12 and 9 times, respectively. The 3D and secondary structural elements of IDUA (Figure 10A,B) show a substantial prevalence of alpha-helices but a dominant occurrence of beta-strands for pathogenic variants. The ACC (Figure 10C) shows that the pathogenic variants have a significantly lower value compared with benign variants, indicating that most pathogenic mutations are far from the protein surface. The residues across IDUA are associated with MTR3D-AF2 scores which range from 1 to 0.6 (Figure 10D). While MTR3D-AF2 scores suggest that many residues from 95 to 150 are somewhat intolerant to missense mutations, this specific region harbors a significantly higher number of ClinVar variants categorised as benign with 14 entries, in comparison to those classified as pathogenic with 61 records. Among the phenotype records, Hurler syndrome is associated with 36 variant entries, while Mucopolysaccharidosis presents a more prevalent association with IDUA [41], observed in 94 records, which shows the substantial implication of IDUA mutations in Mucopolysaccharidoses.

Upon investigating the molecular characteristics of IDUA pathogenic mutations (*n* = 61) in comparison to benign variants (*n* = 14), our analysis reveals that pathogenic mutations are significantly closer to the protein core (*p*-value = 8.02 × 10^−5^), with a median ACC of 3 Å for pathogenic mutations and 19 Å for benign variants. Furthermore, pathogenic variants are closer to active sites (median of 24.59 Å vs. 31.89 Å for benign variants) and to the binding sites (median of 25.95 Å vs. 32.8 Å for benign variants), but it is not statistically significant (*p*-value = 0.076 and 0.068, respectively). In the domain of structural dynamics, pathogenic mutations are associated with regions of increased rigidity (NMA of 0.078 Å^2^ vs. 0.047 Å^2^ for benign variants), albeit without a significant difference (*p*-value = 0.078). Additionally, pathogenic variants are found in areas with decreased flexibility (NMA of 0.033 Å^2^ vs. 0.085 Å^2^ for benign variants) compared with regions housing benign variants (*p*-value = 0.017). Pathogenic variants exhibit higher evolutionary conservation and a lower tolerance to change (r4s *p*-value = 3.04 × 10^−5^), with medians of 0.325 for pathogenic variants versus 2.527 for benign variants. Pathogenic mutations are also linked to decreased stability (mCSM *p*-value = 0.379), with medians of −0.887 for pathogenic variants against −0.726 for benign variants, aligning with the understanding that pathogenic mutations tend to compromise the protein structural and functional integrity.

### 3.7. Lysosomal Acid Glucosylceramidase, GBA1

GBA1 is responsible for encoding the enzyme glucocerebrosidase, which plays a fundamental role in the lysosomal degradation of glucocerebroside into glucose and ceramide. Its activity is centred around two active sites at amino acid positions 274 as a proton donor and 379 as a nucleophile [42]. The enzymatic activity is regulated through interactions with various proteins, including saposin-C, which is known to enhance its membrane association and facilitate substrate binding. GBA1 also interacts with SCARB2, TCP1 and potentially with SNCA; the latter may inhibit glucocerebrosidase activity [43]. Additionally, interaction with GRN prevents aggregation of the GBA1-SCARB2 complex, particularly under stress [44]. GBA1 is in the lysosome membrane, and its function is achieved through an alternative MPR-independent mechanism via SCARB2 [45]. The subunit structure involves interactions that suggest a complex assembly that can form functional units beyond a monomeric state, which may include homodimers or other oligomeric forms.

**Figure 11:**
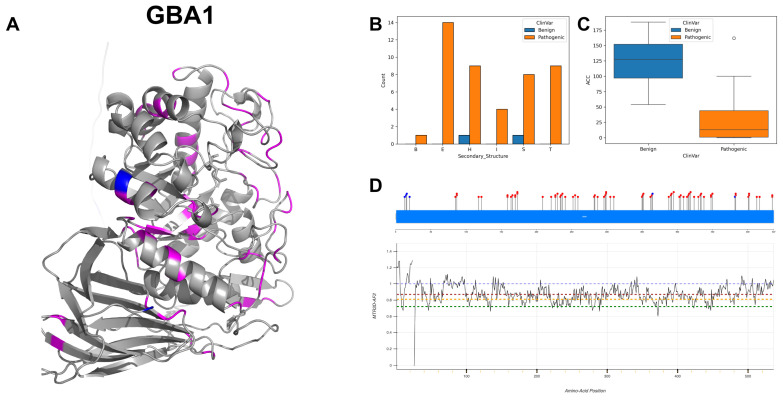
Structural analysis of the GBA1 protein. (**A**) Localisation of benign variants in blue and pathogenic variants in magenta. (**B**) Annotated secondary structure variant mapping. (**C**) ACC distribution. (**D**) Assessment of ClinVar missense variants is presented in a lollipop plot, with benign variants in blue and pathogenic variants in red. The impact of these variants’ tolerance is evaluated through MTR3D-AF2, with green, yellow, red, and blue denoting the 5th, 25th, 50th, and neutrality (MTR = 1) percentiles, respectively.

GBA1 exhibits a spectrum of allelic frequencies and allelic diversity, reflecting the extensive mutational repertoire within this genomic locus. With 91 documented pathogenic and 6 benign variants in ClinVar, GBA1 at the DNA level shows balanced transition and transversion mutations, with 59% GC content to AT content variants including 25 variants from cytosine to thymine and 23 variants from guanine to adenine. The 3D and secondary structure visualisations (Figure 11A,B) demonstrate that pathogenic variants predominantly occur on extended strands and exhibit a lower median ACC value (Figure 11C), indicating that these variants are more buried within the protein structure compared to benign ones. The majority of MTR3D-AF2 scores across GBA1 are in the 0.9 to 0.7 region (Figure 11D), indicating a consistent and small amount of missense variation depletion associated with the gene. The MTR3D-AF2 scores exhibit minimal fluctuation throughout the protein, suggesting that pathogenic variants are unlikely to be particularly overrepresented in specific areas. Among the benign ClinVar variants, four out of the six are situated within a segment characterised by some of the highest MTR3D-AF2 scores in the gene, specifically spanning residues 13 to 23. Additionally, these variants demonstrate effects on the loss or decreasing glucosylceramidase in multiple instances [46], potentially influencing enzymatic efficiency and involvement in the Gaucher disease [47,48]. Apart from Gaucher disease, the variants of GBA1 have also been found in Parkinson’s disease [49,50].

In exploring the molecular characteristics of GBA1, pathogenic mutations (*n* = 67) versus benign variants (*n* = 6), the studied results suggest that pathogenic GBA1 variants are more evolutionarily conserved and are predicted to have a more significant deleterious effect on protein function compared to benign variants. Pathogenic variants have a median average distance to active sites of 24.12. Pathogenic variants have a median deformation NMA of 0.049 and a median fluctuation of 0.014. Pathogenic variants have a median r4s score of 0.857, which indicates higher conservation compared to benign variants with a median r4s score of 1.5869, and the difference is statistically significant (*p*-value = 0.018). Pathogenic variants are predicted to have a more significant impact on protein function with a median mCSM score of −0.996 compared to benign variants with a median of −0.453, and the difference is statistically significant (*p*-value = 0.038).

### 3.8. Putative Bifunctional UDP-N-Acetylglucosamine Transferase and Deubiquitinase ALG13, ALG13

ALG13 encodes an enzyme that is instrumental in protein glycosylation. Specifically, ALG13 is characterised by three active sites at amino acid positions 239, 242 as a nucleophile and 345 for deubiquitinase activity, which is an important regulatory mechanism, including protein degradation, signal transduction and cell cycle control. ALG13 encodes two isoforms with distinct functionalities and implications for disease through alternative splicing: the long isoform 1 (ALG13-is1) and the short isoform 2 (ALG13-is2). Both isoforms share an identical N-terminal region, which contains the catalytic domain responsible for their glycosyltransferase activity. The enzymatic process of eukaryotic N-linked glycosylation within the ER hinges on the action of a UDP-N-acetylglucosamine transferase (GnTase) [51] which itself is composed of ALG13 and ALG14 subunits. This enzyme, which lacks a transmembrane region, is associated with the ER via interaction with the ALG14 protein. ALG14 contains a transmembrane domain, anchoring the complex in the ER membrane [52]. The critical interaction between these subunits is essential for the activity of UDP-GlcNAc transferase, indicating that the assembly of the ALG13 ALG14 complex plays a vital role in controlling N-glycosylation processes [53]. The isoforms diverge significantly in their C-terminal regions. ALG13-is1 possesses ubiquitinase activity in its C-terminal region, implicating it in protein modification processes beyond glycosylation. Mutations in this isoform have been linked to a range of disorders, including congenital disorders of glycosylation type I [54], epileptic encephalopathies [55] and nonsyndromic intellectual disability [56]. In contrast, the C-terminal region of ALG13-is2 facilitates the protein’s localisation to the ER by enabling its interaction with Alg14, a partnership that is vital for its glycosyltransferase function [57]. In a recent study, Wang et al. [58] claimed the finding that, diverging from the previous literature, only the shorter human ALG13 isoform 2, not the longer isoform 1, forms a functional complex with ALG14 that is involved in lipid-linked oligosaccharide synthesis, as isoform 1 of ALG13 does not interact with ALG14 and consequently lacks GnTase activity. Notably, a missense mutation (p.T141L) in ALG13-is2 has been associated with focal segmental glomerulosclerosis and posterior cortical cataract disease [59], indicating its potential impact on renal filtration and eye health. This finding suggests that ALG13-is2 might play a significant role in modifying renal filtration defects, potentially through its involvement in the N-linked glycosylation of podocyte proteins.

**Figure 12:**
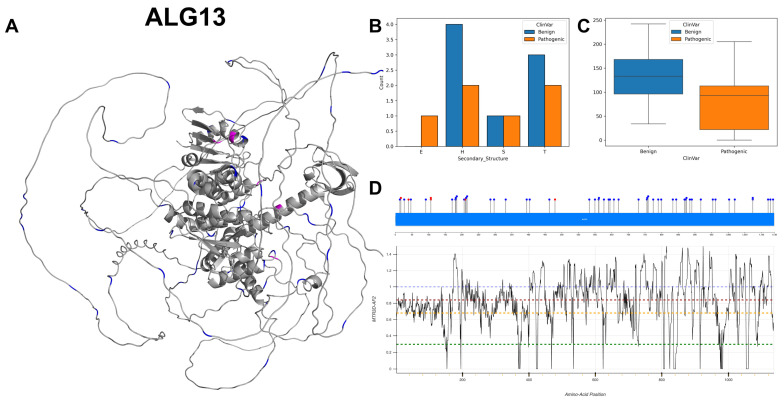
Structural analysis of the ALG13 protein. (**A**) Localisation of benign variants in blue and pathogenic variants in magenta. (**B**) Annotated secondary structure variant mapping. (**C**) ACC distribution. (**D**) Assessment of ClinVar missense variants is presented in a lollipop plot, with benign variants in blue and pathogenic variants in red. The impact of these variants’ tolerance is evaluated through MTR3D-AF2, with green, yellow, red, and blue denoting the 5th, 25th, 50th, and neutrality (MTR = 1) percentiles, respectively.

The structural and molecular analysis of ALG13 is centred around ClinVar records, specifically targeting isoform 1 unless specified otherwise. Amongst the documented mutations of ALG13, transitions predominate, with “A to G” being the most common, closely followed by “C to T” and “T to C”. The gene’s GC content stands at 53.05%, indicating a moderately balanced proportion of G and C nucleotides, which may suggest a stable DNA structure. The 3D and secondary structure visualisation (Figure 12A,B) reveal an abundance of extended strands and alpha-helices for both benign and pathogenic variants, corroborated by ACC (Figure 12C) that indicate the mutated residues are less buried. Although the MTR3D-AF2 scores for residues suggest missense intolerance throughout the gene (Figure 12D), it is noteworthy that only a small number of pathogenic ClinVar variants have been identified. Furthermore, significant fluctuations in scores observed beyond residue 170 could partly be attributed to reduced confidence in the AlphaFold2 structure predictions. Specific mutations at positions 94 and 107, resulting in amino acid substitutions, have been linked to developmental and epileptic encephalopathy (DEE) [55], suggesting a glycosylation defect and reduced enzyme activity.

In exploring the molecular features of ALG13 pathogenic mutations (*n* = 6) as opposed to benign variants (*n* = 50), it is observed that pathogenic mutations are considerably closer to the protein core (*p*-value = 0.505). The median ACC for pathogenic mutations is 36.5 Å, much reduced from the 118 Å median ACC for benign variants. Moreover, pathogenic mutations exhibit a slightly closer distance to active sites (median of 33.33 Å vs. 56.20 Å for benign variants), although this difference does not achieve statistical significance (*p*-value = 0.215). In the analysis of structural dynamic, we find that regions harbouring pathogenic variants are more rigid compared to benign variants (*p*-value of 1.34 × 10^−2^) with a median NMA of 0.002 Å^2^ vs. 0.018 Å^2^ for benign variants. Regions housing pathogenic variants demonstrate a decrease in flexibility (*p*-value = 2.18 × 10^−3^), where the median NMA is 0.0032 Å^2^ for pathogenic variants, substantially lower than the 0.242 Å^2^ observed for benign variants. Pathogenic variants might not be more evolutionarily conserved (r4s *p*-value = 0.165), comparing a median of 0.994 for pathogenic variants to 1.645 for benign variants. Stability analysis indicates that pathogenic variants are less stable (mCSM *p*-value = 0.020), where the median mCSM is −1.566 for pathogenic variants and −0.373 for benign variants.

### 3.9. Exostosin-1, EXT1

EXT1 encodes the enzyme exostosin-1, which is central to the biosynthesis of heparan sulfate, a glycosaminoglycan that plays a critical role in cell signalling. The active site at amino acid position 654 is essential for facilitating the transfer of sugar moieties to the growing polysaccharide chain. EXT1 has multiple binding sites for UDP-N-acetyl-alpha-D-glucosamine, a substrate for the glycosyltransferase reaction. The presence of binding sites at residues 440, 549, 565, 566, 567, 653, 654 and 701 suggests a complex interaction necessary for the enzyme’s function. Notably, the residue at position 440 is identified as a benign variant in ClinVar records. EXT1 forms a homo/heterooligomeric complex with EXT2 [60], another glycosyltransferase involved in heparan sulfate biosynthesis, as well as with NDST1, an N-deacetylase/N-sulfotransferase. EXT1’s subcellular location is within the endoplasmic reticulum and Golgi apparatus membranes. The topological domain and transmembrane regions of EXT1 indicate it is a type II membrane protein with a single-pass transmembrane helix [61], positioning the catalytic domain within the lumen of the ER and Golgi.

**Figure 13:**
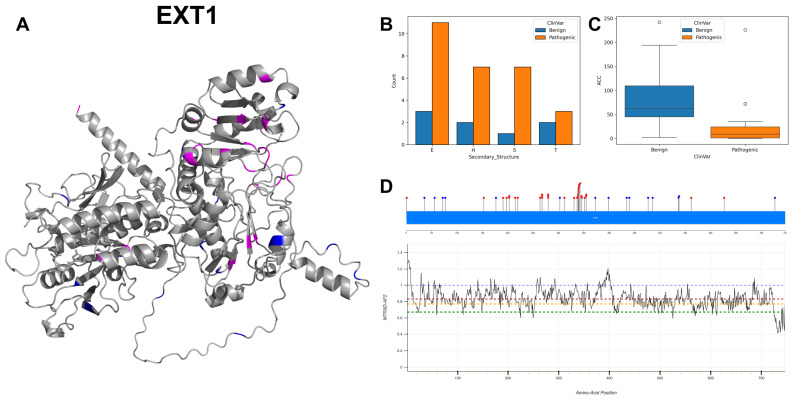
Structural analysis of the EXT1 protein. (**A**) Localisation of benign variants in blue and pathogenic variants in magenta. (**B**) Annotated secondary structure variant mapping. (**C**) ACC distribution. (**D**) Assessment of ClinVar missense variants is presented in a lollipop plot, with benign variants in blue and pathogenic variants in red. The impact of these variants’ tolerance is evaluated through MTR3D-AF2, with green, yellow, red, and blue denoting the 5th, 25th, 50th, and neutrality (MTR = 1) percentiles, respectively.

EXT1 has recorded a spectrum of nucleotide variants, predominantly characterised by G to A transitions, with 12 instances, followed by A to G and G to T mutations at 7 and 5 counts, respectively. The 3D structure and secondary structure histogram (Figure 13A,B) visualises a marginally more pronounced presence of extended strands compared to other structures, accompanied by a substantial difference in the ACC values of pathogenic and benign variants (Figure 13C). The majority of residues across EXT1 are associated with missense intolerance scores (MTR3D-AF2) ranging from greater than 1 to 0.6 (Figure 13D). Unexpectedly, there are no pathogenic variants within the segment most depleted of missense variants, specifically from residues 722 to 747. Instead, there is a significant cluster of pathogenic variants found between residues 310 to 360, a region characterised by moderate intolerance. This gene exhibits a total of 75 variants, the majority of which (61) are pathogenic, while 14 variants are considered benign. In terms of phenotype records, Multiple Congenital Exostoses prominently appear in 44 instances related to EXT1 [62], suggesting a potential association of these mutations with this specific phenotype.

In the evaluation of molecular characteristics of EXT1 pathogenic mutations (*n* = 34) in contrast with benign variants (*n* = 15), our analysis reveals that pathogenic mutations are significantly nearer to the protein core (*p*-value = 1.98 × 10^−2^) with the median ACC of 6 Å vs. 35 Å for benign variants. Further analysis shows that pathogenic variants are unexpectedly farther away from both active sites (*p*-value = 2.31 × 10^−3^) and binding sites (*p*-value = 1.7 × 10^−3^), with the median distance to active sites of 48.89 Å vs. 22.98 Å for benign variants and median distance to binding sites of 54.55 Å vs. 21.68 Å for benign variants. In examining the structural dynamics, pathogenic variants are found in regions that are more rigid (NMA of 0.153 Å^2^ versus 0.061 Å^2^ for benign variants), though this difference does not reach statistical significance (*p*-value = 4.39 × 10^−2^). In terms of flexibility, regions containing pathogenic mutations display reduced flexibility (*p*-value = 5.08 × 10^−3^), with a median NMA of 0.058 Å^2^ to 0.1838 Å^2^ for benign variants. When considering evolutionary conservation, pathogenic EXT1 variants are more conserved and less tolerant to change than benign variants (r4s *p*-value = 5.39 × 10^−4^), with median values of 0.624 vs. 1.649 for benign variants. Regarding stability, pathogenic variants are associated with a decrease in stability (mCSM *p*-value = 6.21 × 10^−3^), with median values of −0.82 vs. −0.42 for benign variants.

### 3.10. Beta-Hexosaminidase Subunit Alpha, HEXA

HEXA encodes the alpha subunit of beta-hexosaminidase, an enzyme that plays a vital role in the degradation of GM2 gangliosides in lysosomes. A critical active site at position 323 acts as a proton donor [63], suggesting its essential role in the catalytic mechanism of HEXA. The subunit structure of HEXA has three identified beta-hexosaminidase isozymes: isozyme A is a heterodimer composed of one alpha and one beta subunit, isozyme B is a homodimer of two beta subunits, and isozyme S is a homodimer of two alpha subunits, which influences the substrate specificity of the alpha subunit active site [64]. The subcellular location of HEXA is within the lysosome, which is consistent with its role in the catabolism of glycolipids.

**Figure 14:**
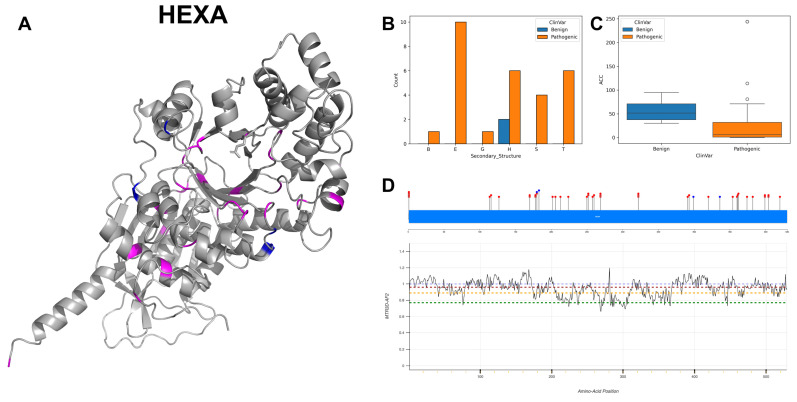
Structural analysis of the HEXA protein. (**A**) Localisation of benign variants in blue and pathogenic variants in magenta. (**B**) Annotated secondary structure variant mapping. (**C**) ACC distribution. (**D**) Assessment of ClinVar missense variants is presented in a lollipop plot, with benign variants in blue and pathogenic variants in red. The impact of these variants’ tolerance is evaluated through MTR3D-AF2, with green, yellow, red, and blue denoting the 5th, 25th, 50th, and neutrality (MTR = 1) percentiles, respectively.

HEXA exhibits a dominant frequency of G to A mutations, totalling 15 instances, with C to T mutations noted on 7 occasions. The presented 3D structure (Figure 14A) and secondary structure (Figure 14B) indicate that benign variants predominantly occur within alpha-helices, whereas pathogenic variants are more prevalent amongst beta-strands, alpha-helices and turns. The ACC values (Figure 14C) for both benign and pathogenic variants show comparable ranges, although pathogenic variants are noted to be situated closer to the protein’s core. The majority of HEXA residues are linked with missense intolerance scores (MTR3D-AF2) between greater than 1 and 0.8 (Figure 14D), suggesting minimal missense intolerance throughout the gene. ClinVar records a total of 45 variants related to HEXA, including 41 pathogenic and 4 benign variants, with 42 of the pathogenic variants being associated with Tay–Sachs disease [65].

In exploring the molecular characteristics of HEXA pathogenic mutations (*n* = 41) versus benign variants (*n* = 4), our analysis reveals that pathogenic mutations are significantly closer to the protein core (*p*-value = 2.79 × 10^−2^) with a median ACC of 22 Å vs. 62 Å for benign variants. Further investigation reveals that pathogenic variants are slightly closer to the active sites (median of 23.20 Å vs. 27.92 Å for benign variants) compared to benign variants. However, this proximity does not reach statistical significance (*p*-value = 0.129). When assessing structural dynamics, pathogenic variants are found in more rigid regions (median NMA of 0.081 Å^2^ vs. 0.254 Å^2^ for benign variants), yet this observed increase in rigidity is not significantly different (*p*-value = 0.165). Similarly, pathogenic variants appear in regions that are slightly more flexible (median NMA of 0.070 Å^2^ vs. 0.046 Å^2^ for benign variants). Again, this difference in flexibility is not statistically significant (*p*-value = 0.843). In terms of evolutionary conservation, pathogenic variants exhibit a trend towards higher conservation (media of 1.375 vs. 2.817 for benign variants), but not achieving statistical significance (r4s *p*-value = 0.202). Regarding stability, pathogenic variants tend to be less stable (median of −0.425 vs. −1.208 for benign variants), but the difference in stability does not reach statistical significance (mCSM *p*-value = 0.281).

### 3.11. Bifunctional UDP-N-Acetylglucosamine 2-Epimerase/N-Acetylmannosamine Kinase, GNE

GNE encodes the enzyme UDP-N-acetylglucosamine 2-epimerase/N-acetylmannosamine kinase, which is key in the biosynthesis of sialic acid, an essential component of glycoproteins and glycolipids. The active site at position 517 is implicated in substrate binding and catalysis [66], which is vital for its role in the sialylation process. Binding sites for ATP and substrate, spanning residues 411 to 420, 477, 489, 517 and 543 to 552, highlight its need for ATP in its kinase domain and its interaction with substrates in the epimerase domain. Particularly notable is the span of 411 to 420, which suggests a region of interaction for ATP binding, fundamental for the kinase activity of GNE. The zinc binding sites of GNE at residues 569, 579, 581 and 586 indicate a structural or catalytic role for this metal ion. The subunit structure consists of homodimers and homohexamers, reflecting the potential for allosteric effects. Its subcellular location in the cytoplasm aligns with its involvement in the cytosolic steps of sialic acid synthesis before its transfer to the Golgi apparatus.

**Figure 15:**
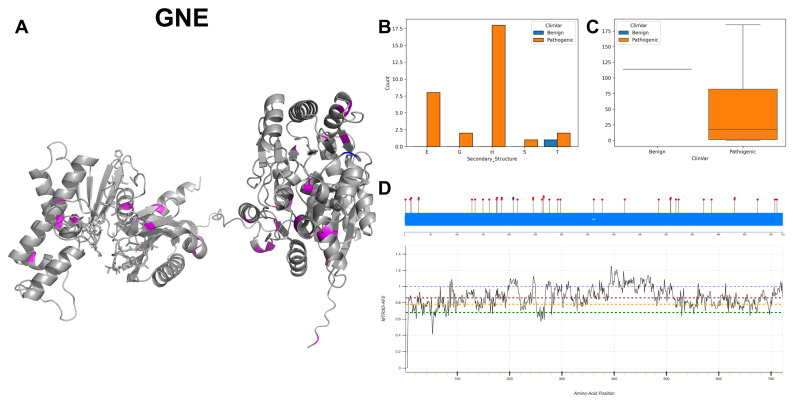
Structural analysis of the GNE protein. (**A**) Localisation of benign variants in blue and pathogenic variants in magenta. (**B**) Annotated secondary structure variant mapping. (**C**) ACC distribution. (**D**) Assessment of ClinVar missense variants is presented in a lollipop plot, with benign variants in blue and pathogenic variants in red. The impact of these variants’ tolerance is evaluated through MTR3D-AF2, with green, yellow, red, and blue denoting the 5th, 25th, 50th, and neutrality (MTR = 1) percentiles, respectively.

The DNA coding variants of GNE exhibit a spectrum of mutations, with a notable prevalence of 11 C to T mutations, 9 instances of T to C mutations and 7 G to A mutations. For 3D and secondary structures (Figure 15A,B), the documented pathogenic variants predominantly occur within the alpha-helices. Given that most recorded variants are pathogenic, there is a comparatively broad range of ACC values (Figure 15C), indicating increased solvent accessibility due to the 3D structure. The residues across GNE are associated with MTR3D-AF2 scores ranging from 1 to 0.6 (Figure 15D), suggesting a small yet consistent level of missense intolerance throughout the gene. A notable absence of variation is observed in residues 1 to 5, where only a single pathogenic ClinVar variant is found. In total, GNE is associated with 39 variants, comprising 38 pathogenic and a single benign variant. Most of these pathogenic variants are implicated in its myopathy [67], while numerous other records are linked to sialuria [68].

In the exploration of GNE’s pathogenic mutations (*n* = 38) versus benign variants (*n* = 1), the results suggest that the pathogenic variants of GNE have a recorded median ACC of 8.0 Å, which might suggest alterations in protein surface characteristics relevant to function or interactions. A median distance to active sites for pathogenic variants is given as 29.48 Å, and the median distance to binding sites is given as 28.48 Å. Regarding structural dynamics, the rigidity of regions housing pathogenic variants shows a median scaled deformation NMA of 0.036 Å^2^ for pathogenic variants. The regions hosting pathogenic variants also show a median scaled fluctuation NMA of 0.103 Å^2^, indicative of their dynamic behaviours. The median r4s score for pathogenic variants is 0.437, indicating evolutionary conservation at these sites. The predicted impact on protein function for pathogenic variants, as measured by mCSM, is notably deleterious with a score of −1.742, suggesting these mutations are likely to adversely affect the function of GNE.

### 3.12. Adenine DNA Glycosylase, MUTYH

MUTYH is implicated in the base excision repair pathway, coding for the DNA glycosylase enzyme, which recognises and excises oxidatively damaged DNA bases. Notably, active site 131, as a proton donor/acceptor, is essential for the catalysis and repair of DNA lesions. The binding sites at residues 287, 294, 297 and 303 suggest a critical role for this metallic cluster in facilitating the redox reactions during the DNA repair process, while residue 233 is noted as a transition state stabiliser, indicating its possible role in stabilising the enzyme–substrate complex [69]. MUTYH localises to both the nucleus and mitochondrion, highlighting its role in safeguarding the integrity of both nuclear and mitochondrial DNA.

**Figure 16:**
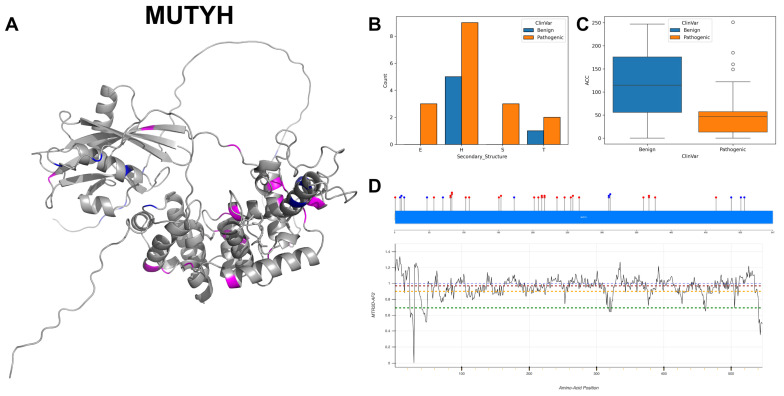
Structural analysis of the MUTYH protein. (**A**) Localisation of benign variants in blue and pathogenic variants in magenta. (**B**) Annotated secondary structure variant mapping. (**C**) ACC distribution. (**D**) Assessment of ClinVar missense variants is presented in a lollipop plot, with benign variants in blue and pathogenic variants in red. The impact of these variants’ tolerance is evaluated through MTR3D-AF2, with green, yellow, red, and blue denoting the 5th, 25th, 50th, and neutrality (MTR = 1) percentiles, respectively.

MUTYH exhibits a relatively balanced transition from GC to AT content, including 10 instances of G to A mutations, 7 instances of C to T, 6 instances of A to G and 4 of C to G, highlighting nuanced alterations in nucleotide composition. The visualisation of the 3D structure and secondary structure (Figure 16A,B) reveals that pathogenic variants primarily occur in alpha-helices and turns, whilst benign variants predominantly appear in alpha-helices. The ACC boxplot (Figure 16C) demonstrates a significant difference between benign and pathogenic variants, with the latter showing a markedly lower median ACC value. The majority of residues across MUTYH are associated with MTR3D-AF2 scores ranging from greater than 1 to 0.9 (Figure 16D), interspersed with small segments characterised by severe missense intolerance. The distribution of ClinVar variants across the gene does not seem to correspond with the MTR3D-AF2 scores. Variations within MUTYH total 38 variants, comprising 26 pathogenic and 12 benign variants. The link of MUTYH with Familial Adenomatous Polyposis [70] and Hereditary Cancer-Predisposing Syndrome [71] shows its importance in the realm of hereditary cancer susceptibility.

In analysing the molecular characteristics of MUTYH pathogenic mutations (*n* = 26) versus benign variants (*n* = 12), pathogenic mutations exhibit a median ACC of 55 Å, indicating that pathogenic variants are relatively exposed on the protein surface. The median distance of pathogenic variants to active sites is 28.25 Å, while the median distance to binding sites is 27.67 Å, and the standard deviation in these distances is 3.23, which may suggest that the pathogenic variants could potentially influence the activity of the enzyme without directly disrupting the active or binding site. In terms of protein dynamics, regions that host pathogenic variants are associated with a median scaled deformation of 1.98 × 10^−3^ Å^2^ and a median scaled fluctuation NMA of 8.66 × 10^−5^ Å^2^. These values suggest subtle differences in the structural flexibility and rigidity of regions harbouring pathogenic mutations. The median r4s score for pathogenic variants is 2.32, indicating evolutionary conservation at these sites. The predicted impact on protein function for pathogenic variants, as measured by mCSM, is notably deleterious with a score of −1.41, suggesting these mutations are likely to adversely affect the protein’s structure or function.

### 3.13. Lysosomal Alpha-Mannosidase, MAN2B1

MAN2B1 encodes lysosomal alpha-mannosidase, an enzyme crucial in the degradation of glycoproteins. Its active site at position 196, acting as a nucleophile, plays an essential role in the hydrolysis of the mannosyl linkages in glycoproteins. Its binding sites at residues 72, 74, 196 and 446 for Zn (2+) ions suggest that these metal ions are key cofactors for enzymatic activity [72]. The presence of multiple Zn (2+) binding sites indicates a reliance on these ions for enzymatic function, possibly impacting the conformational dynamics of the enzyme. The localisation to the lysosome is typical for enzymes involved in the degradation processes, affirming its role in cellular catabolism.

**Figure 17:**
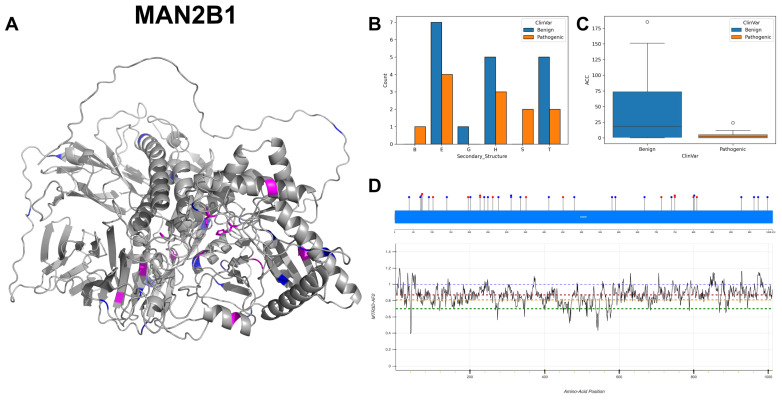
Structural analysis of the MAN2B1 protein. (**A**) Localisation of benign variants in blue and pathogenic variants in magenta. (**B**) Annotated secondary structure variant mapping. (**C**) ACC distribution. (**D**) Assessment of ClinVar missense variants is presented in a lollipop plot, with benign variants in blue and pathogenic variants in red. The impact of these variants’ tolerance is evaluated through MTR3D-AF2, with green, yellow, red, and blue denoting the 5th, 25th, 50th, and neutrality (MTR = 1) percentiles, respectively.

MAN2B1 exhibits a range of nucleotide coding variants, including eight occurrences of G to A mutations, seven instances of C to T, five changes from G to T and five changes from C to G, encompassing both transitions and transversions. The 3D structure and secondary structure (Figure 17A,B) demonstrate a notable abundance of alpha-helices and extended strands, reflective of its functional conformation within the lysosomal environment. Regarding the ACC (Figure 17C), pathogenic variants present a significantly reduced median value and a narrower range compared to benign variants. The residues across MAN2B1 are associated with MTR3D-AF2 scores ranging from 1 to 0.6 (Figure 17D), indicating a small yet consistent level of missense intolerance throughout the gene. This level of intolerance corresponds with the ratio of benign to pathogenic ClinVar variants identified within the gene. The documented variants of MAN2B1 collectively amount to 36, with 14 classified as pathogenic and 22 as benign. The prevalence of mutations in the MAN2B1 gene is consistent with the clinical manifestation of Alpha-Mannosidosis [73].

In exploring the pathogenic mutations (*n* = 14) versus benign variants (*n* = 22) of the MAN2B1 gene, the studied results suggest that pathogenic variants in MAN2B1 are characterised by closer proximity to both active and binding sites, and they are more evolutionarily conserved. The median ACC for pathogenic mutations is marginally higher at 1 Å compared to 0.5 Å for benign variants, suggesting minimal and not significant distinction in terms of spatial accessibility between the two groups (ACC *p*-value = 0.91). Pathogenic variants are significantly closer to the active sites (median distance of 17.08 Å) compared to benign variants (median distance of 23.38 Å), which may affect enzymatic activity (*p*-value = 0.037). Pathogenic variants are significantly closer to binding sites (median distance of 18.65 Å) than benign variants (median distance of 26.14 Å), potentially impacting substrate binding and overall protein function (*p*-value = 0.018). No significant difference in deformation from normal mode analysis is observed between regions hosting benign and pathogenic variants (*p*-value = 0.205). Fluctuations from normal mode analysis do not significantly differ between benign and pathogenic variants (*p*-value = 0.356). Pathogenic variants are more evolutionarily conserved with a lower median r4s score (0.435) than benign variants (1.08). There is no significant difference in the predicted impact of mutations on protein function between benign (median mCSM of −1.197) and pathogenic (median mCSM of −1.223) variants (*p*-value = 0.918).

### 3.14. Beta-Hexosaminidase Subunit Beta, HEXB

HEXB encodes the beta subunit of beta-hexosaminidase, an enzyme that is key in the degradation of GM2 gangliosides. The active site of HEXB, found at position 355, acts as a proton donor which is crucial for catalysing the hydrolysis of the terminal beta-N-acetylhexosamines in GM2 gangliosides. HEXB exists in several forms: hexosaminidases A (Hex-A), B (Hex-B) and S (Hex-S), each with different subunit compositions and substrate specificities. The lack of glycosylation at site 497 is noted and could have implications for the function and processing of HEXB.

**Figure 18:**
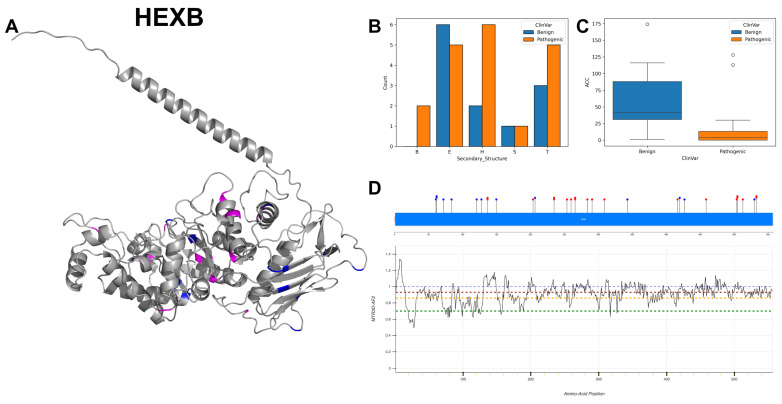
Structural analysis of the HEXB protein. (**A**) Localisation of benign variants in blue and pathogenic variants in magenta. (**B**) Annotated secondary structure variant mapping. (**C**) ACC distribution. (**D**) Assessment of ClinVar missense variants is presented in a lollipop plot, with benign variants in blue and pathogenic variants in red. The impact of these variants’ tolerance is evaluated through MTR3D-AF2, with green, yellow, red, and blue denoting the 5th, 25th, 50th, and neutrality (MTR = 1) percentiles, respectively.

HEXB displays a wide range of nucleotide variations, with C to T alterations occurring most frequently, followed by A to G and G to A changes. The secondary structure (Figure 18B) profile is dominated by alpha-helices and beta-strands, confirmed with the 3D structure visualisation (Figure 18A), suggesting that the structural configuration may impact its catalytic activity. The ACC reveals that pathogenic variants have significantly lower values (Figure 18C), suggesting these variants are more buried compared to benign ones. Residues across HEXB are associated with MTR3D-AF2 scores ranging from 1 to 0.6 (Figure 18D), although the majority of scores are around or above 1. The ClinVar record documents a total of 33 variants for HEXB, with 13 classified as benign and 20 as pathogenic. Sandhoff disease [74] is notably associated with 31 of these genetic alterations.

In exploring the molecular characteristics of HEXB pathogenic mutations (*n* = 20) versus benign variants (*n* = 13), our analysis reveals that pathogenic variants have a significantly lower (*p* = 5.15 × 10^−3^) median accessible surface area (median ACC of 0.0 Å) compared to benign variants (median ACC of 35.0 Å), which suggests that pathogenic variants are likely buried within the protein structure. There is no statistically significant difference in the average distance to active sites between benign and pathogenic variants, with pathogenic variants showing a median distance of 22.08 compared to benign variants at 38.07 (*p*-value = 0.254). No significant difference in median deformation from normal mode analysis is observed between regions housing benign and pathogenic variants (*p*-value = 0.330). Both regions that hold benign and pathogenic variants show similar median fluctuations from NMA, with no significant difference (*p*-value = 0.802). HEXB are significantly more conserved (*p*-value = 3.5 × 10^−4^) for pathogenic variants (median r4s score of 0.380) than benign variants (median r4s score of 1.804). There is no significant difference in the predicted impact of mutations on protein function between benign (median mCSM of −0.914) and pathogenic variants (median mCSM of −1.195) of HEXB (*p*-value = 0.282).

### 3.15. 1,4-Alpha-Glucan-Branching Enzyme, GBE1

GBE1 codes for the enzyme glycogen branching enzyme 1, adding alpha-1,6 linked branches to the growing glycogen molecule, which is integral to the process of glycogen biosynthesis, thereby increasing the solubility and bioavailability of glycogen. The active sites at amino acid positions 357 and 412 are crucial for the enzyme’s catalytic activity, with position 357 serving as the nucleophile and 412 as the proton donor. The binding regions spanning residues 62–63, 91–93, 118–121 and 333–336 are associated with substrate interaction, suggesting a complex substrate binding domain necessary for its proper function. GBE1 functions as a monomer, and the subcellular location of GBE1 is in the cytoplasm where glycogen synthesis occurs [75].

**Figure 19:**
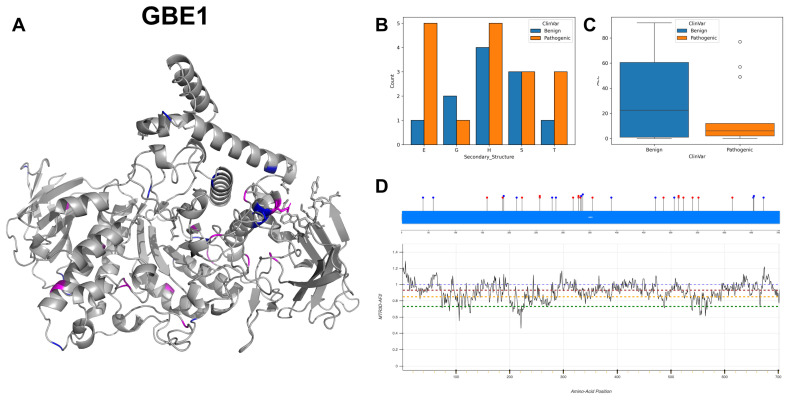
Structural analysis of the GBE1 protein. (**A**) Localisation of benign variants in blue and pathogenic variants in magenta. (**B**) Annotated secondary structure variant mapping. (**C**) ACC distribution. (**D**) Assessment of ClinVar missense variants is presented in a lollipop plot, with benign variants in blue and pathogenic variants in red. The impact of these variants’ tolerance is evaluated through MTR3D-AF2, with green, yellow, red, and blue denoting the 5th, 25th, 50th, and neutrality (MTR = 1) percentiles, respectively.

GBE1 exhibits a range of mutations, notably characterised by G to A and A to G alterations, along with T to C and C to T changes. The secondary structure (Figure 19B) displays a predominance of alpha-helices and extended strands, interspersed with fewer turns and helices, potentially linked to GBE1’s role in glycogen branching. In contrast to pathogenic variants, which show a lower ACC indicative of reduced accessibility, benign variants demonstrate a wider value range (Figure 19C). The 3D structure of GBE1 (Figure 19A) also shows that most of the benign variants are exposed. The majority of residues across GBE1 are associated with MTR3D-AF2 scores ranging from greater than 1 to 0.6 (Figure 19D), indicating a modest yet consistent level of missense intolerance throughout the gene. Of the 31 variants documented in ClinVar for GBE1, 14 are classified as benign and 17 as pathogenic. These findings highlight the genetic intricacies associated with glycogen storage disease [76], emphasising the importance of these specific mutations and their potential implications in disease pathology.

In exploring the molecular characteristics of GBE1 pathogenic mutations (*n* = 17) versus benign variants (*n* = 14), our analysis reveals that the ACC analysis between pathogenic and benign variants does not show a significant difference (*p*-value = 0.870). The median ACC for pathogenic mutations is slightly higher at 7 Å compared to 5.5 Å for benign variants, indicating pathogenic and benign variants are similarly positioned relative to the protein surface. Regarding the average distance to active sites, the median distances are 24.09 Å and 25.70 Å for pathogenic and benign variants, respectively, with no significant difference (*p*-value = 0.492). Similarly, the median distances are 37.68 Å for pathogenic variants and 39.48 Å for benign ones, with no significant difference (*p*-value = 0.793). The structural dynamics, assessed through scaled deformation and fluctuation NMA, also do not exhibit significant differences (*p*-values as 0.635 for deformation and 0.875 for fluctuation, respectively), suggesting that pathogenic and benign variants affect the protein’s structural rigidity and flexibility in similar ways. Evolutionary conservation shows a borderline significant difference (*p*-value of 0.050), with median r4s scores of 0.630 for pathogenic variants and 1.561 for benign variants. This suggests that pathogenic variants tend to occur in more evolutionarily conserved regions. In terms of protein stability, the median mCSM values are −0.146 for pathogenic variants and −1.14 for benign variants, indicating that pathogenic mutations are associated with a lesser impact on protein stability compared to benign variants (*p*-value = 0.048). This is a notable deviation from the general expectation that pathogenic mutations tend to destabilise proteins more significantly.

### 3.16. Phosphatidylinositol N-Acetylglucosaminyltransferase Subunit A, PIGA

PIGA is a crucial component of the glycosylphosphatidylinositol-N-acetylglucosaminyltransferase (GPI-GnT) complex, which plays a fundamental role in the biosynthesis of GPI anchors. The GPI-GnT complex is known to be composed of several components including PIGA, PIGC, PIGH, PIGP, PIGQ, PIGY and DPM2. Specifically, PIGA’s direct interaction with PIGY is noted to regulate the activity of the glycosylphosphatidylinositol-N-acetylglucosaminyltransferase [77]. PIGA is located in the endoplasmic reticulum membrane as a single-pass membrane protein, allowing it to participate in the early steps of GPI anchor synthesis which occurs in the ER. The topological domain of PIGA extends from the cytoplasmic side from residues 1 to 421 and has a lumenal domain from residues 443 to 484. The transmembrane helical region spans residues 422 to 442, anchoring the protein in the membrane.

**Figure 20:**
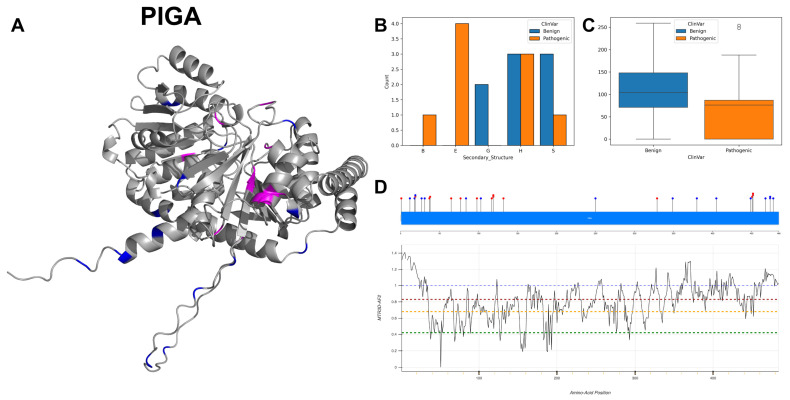
Structural analysis of the PIGA protein. (**A**) Localisation of benign variants in blue and pathogenic variants in magenta. (**B**) Annotated secondary structure variant mapping. (**C**) ACC distribution. (**D**) Assessment of ClinVar missense variants is presented in a lollipop plot, with benign variants in blue and pathogenic variants in red. The impact of these variants’ tolerance is evaluated through MTR3D-AF2, with green, yellow, red, and blue denoting the 5th, 25th, 50th, and neutrality (MTR = 1) percentiles, respectively.

PIGA exhibits a significant frequency of A to G and G to A nucleotide transitions, along with C to T and T to C changes. The visualisation of the 3D structure and secondary structure (Figure 20A,B) indicates a predominance of alpha-helices and extended strands compared to other structural architectures. Pathogenic mutations generally have lower ACC than benign variants (Figure 20C), although the value ranges are comparable. MTR3D-AF2 scores associated with residues across PIGA vary widely (Figure 20D). Benign ClinVar variants tend to cluster at the gene’s extremities, where the greatest variation is observed. Most pathogenic variants are located from residues 50 to 150, a region characterised by very little tolerance to missense mutations as per MTR3D-AF2 scores. PIGA’s association in ClinVar records with multiple congenital anomalies-hypotonia-seizures syndrome type 2 [78] further underscores the potential clinical implications of these mutations in the context of this syndrome.

In exploring the molecular characteristics of PIGA pathogenic mutations (*n* = 15) versus benign variants (*n* = 16), the studied results suggest that pathogenic variants are characterised by a lower accessible surface area and lower fluctuations in normal mode analysis, and they are found at more conserved sites. Pathogenic PIGA variants exhibit a significantly lower median accessible surface area (median ACC of 0.0) compared to benign variants (median ACC of 71.0), suggesting that these mutations may result in residues being more buried within the protein structure (*p*-value = 0.025). The analysis of scaled deformation NMA suggests a non-significant (*p*-value = 0.056) trend towards increased rigidity in regions harbouring pathogenic variants (median of 0.001 Å^2^) compared to those with benign variants (median of 0.038 Å^2^). Conversely, scaled fluctuation NMA shows a significant difference (*p*-value = 0.016), indicating that pathogenic variants are associated with regions of reduced flexibility (median of 7 × 10^−4^ Å^2^ for pathogenic vs. 2.9 × 10^−3^ Å^2^ for benign variants). Pathogenic PIGA variants are more evolutionarily conserved (*p*-value = 4.3 × 10^−4^) with a lower median r4s score (0.603) compared to benign variants (3.196). Pathogenic PIGA variants are predicted to have a more significant impact on protein function (median mCSM score of −1.839) than benign variants (median mCSM score of −0.399), although this difference is not statistically significant (*p*-value = 0.099).

### 3.17. Glycogen Debranching Enzyme, AGL

AGL encodes the glycogen debranching enzyme, critical in glycogenolysis, which is essential for mobilising glycogen reserves. The conserved active sites at amino acid positions 526, 529 and 627 are inferred to catalyse the hydrolysis of alpha-1,6-glucosidic linkages during glycogen degradation. The subcellular location of AGL is primarily in the cytoplasm, consistent with its metabolic role; however, it translocates to the nucleus under conditions that trigger glycogen breakdown [79].

**Figure 21:**
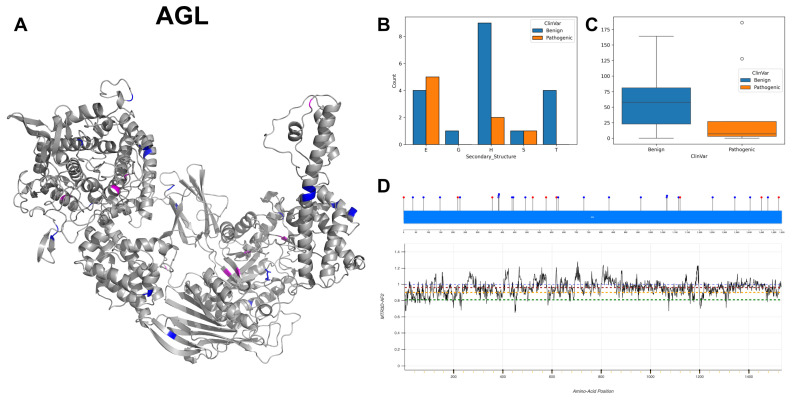
Structural analysis of the AGL protein. (**A**) Localisation of benign variants in blue and pathogenic variants in magenta. (**B**) Annotated secondary structure variant mapping. (**C**) ACC distribution. (**D**) Assessment of ClinVar missense variants is presented in a lollipop plot, with benign variants in blue and pathogenic variants in red. The impact of these variants’ tolerance is evaluated through MTR3D-AF2, with green, yellow, red, and blue denoting the 5th, 25th, 50th, and neutrality (MTR = 1) percentiles, respectively.

AGL demonstrates a comparatively balanced pattern of nucleotide transitions, notably including G to A, A to G and C to T mutations. The visualisation of the 3D and secondary structures (Figure 21A,B) reveals a predominance of extended strands and alpha-helices, indicative of structures crucial for the enzyme’s glycogen debranching function. Pathogenic mutations possess significantly lower ACC compared to benign variants (Figure 21C), suggesting these pathogenic alterations are more internalised within the protein structure. Residues across AGL are associated with MTR3D-AF2 scores predominantly greater than 1, up to 0.8 (Figure 21D), indicating a generally low level of missense intolerance throughout the gene. This is consistent with the observed ratio of benign to pathogenic ClinVar variants. AGL’s documented variants in ClinVar include 21 characterised as benign and 9 as pathogenic, underscoring the enzyme’s influence on glycogen storage disease phenotypes [80,81].

In the exploration of the molecular characteristics of AGL, contrasting pathogenic mutations (*n* = 9) with benign variants (*n* = 21), the studied results suggest that the median distances are closely matched (*p*-value = 0.679) at 40.23 Å for pathogenic variants and 40.97 Å for benign variants. In terms of structural dynamics, assessed through scaled deformation and fluctuation via NMA, no significant differences are observed, with the *p*-values for scaled deformation NMA and scaled fluctuation NMA being 0.206 and 0.129, respectively. These results indicate that pathogenic and benign variants similarly affect the protein’s structural rigidity and flexibility. Evolutionary conservation also does not show a significant difference between pathogenic and benign variants, suggesting a similar evolutionary conservation status (*p*-value = 0.254). The median r4s scores are 0.912 for pathogenic variants and 1.530 for benign variants. The median mCSM values are −0.908 for pathogenic variants and −0.904 for benign variants with no significant difference (*p*-value = 0.953), underscoring those mutations, irrespective of their pathogenicity, have a similar impact on the protein’s stability.

### 3.18. Chitobiosyldiphosphodolichol Beta-Mannosyltransferase, ALG1

ALG1 encodes for an enzyme that is integral to the biosynthesis of oligosaccharides. Its localisation to the ER membrane is indicative of its role in the early stages of the N-linked glycosylation pathway [82]. The topological domain, with a short cytoplasmic segment and a substantial lumenal portion, further supports its involvement in the ER-specific processes. The single-pass type II orientation, featuring an N-terminal transmembrane domain that acts as a signal anchor, is typical of enzymes that remain stably associated with the ER membrane.

**Figure 22:**
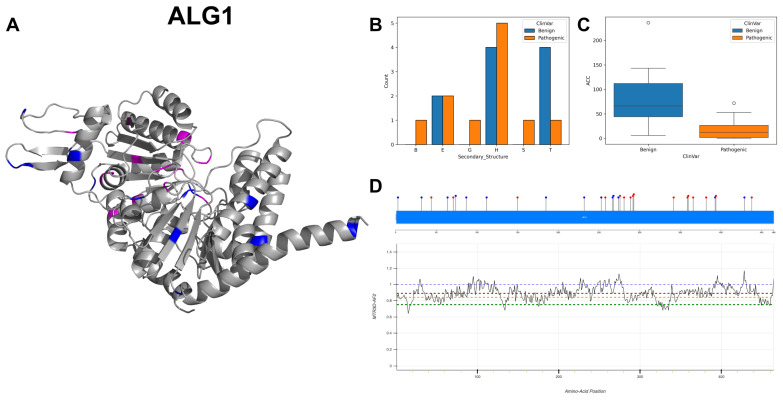
Structural analysis of the ALG1 protein. (**A**) Localisation of benign variants in blue and pathogenic variants in magenta. (**B**) Annotated secondary structure variant mapping. (**C**) ACC distribution. (**D**) Assessment of ClinVar missense variants is presented in a lollipop plot, with benign variants in blue and pathogenic variants in red. The impact of these variants’ tolerance is evaluated through MTR3D-AF2, with green, yellow, red, and blue denoting the 5th, 25th, 50th, and neutrality (MTR = 1) percentiles, respectively.

ALG1 displays a balanced array of nucleotide changes, characterised by transversions and transitions, with relatively equal counts of benign and pathogenic mutations. Specifically, nucleotide variants include seven instances of C to T alterations, four occurrences each of C to G and C to A changes and three instances of G to A variations. Examination of the 3D structure and secondary structural elements (Figure 22A,B) reveals a marked predominance of alpha-helices and beta-turns. The solvent accessibility evaluation indicates that pathogenic variants have a narrower interquartile range and lower median than benign ones (Figure 22C), suggesting they are more internally localised within the protein matrix. The majority of residues across ALG1 are associated with MTR3D-AF2 scores ranging from 1 to 0.7 (Figure 22D), indicating a modest but consistent level of missense intolerance throughout the gene. ALG1 variations are primarily documented in association with congenital disorders of glycosylation [83], underscoring the importance of variants in understanding the molecular basis of these conditions.

In exploring the molecular characteristics of ALG1 pathogenic mutations (*n* = 16) versus benign variants (*n* = 14), our analysis shows that pathogenic variants have a significantly lower median accessible surface area (median ACC of 4.5 Å) compared to benign variants (median ACC of 70.0 Å), which may suggest an increased likelihood of the variants being buried within the protein structure (*p*-value = 0.003). Regions hosting pathogenic variants show significantly lower (*p*-value = 0.01) median fluctuations from normal mode analysis (median fluctuation of 0.001) compared to benign variants (median fluctuation of 0.004). The scaled deformation form NMA does not show a significant difference (*p*-value = 0.903) in the rigidity of regions harbouring pathogenic (median of 0.037 Å^2^) versus benign variants (median of 0.042 Å^2^), suggesting that the presence of pathogenic mutations does not significantly alter the structural rigidity compared to benign variants. Pathogenic variants are significantly more conserved (median r4s score of 0.624) than benign variants (median r4s score of 2.714), indicating that the pathogenic mutations occur at sites critical to protein function (*p*-value = 5.2 × 10^−4^). There is no significant difference in the predicted impact of mutations on protein function between benign and pathogenic variants, with pathogenic variants having a slightly more deleterious median mCSM score (median mCSM of −0.619) compared to benign variants (median mCSM of −0.488), though this difference is not statistically significant (*p*-value = 0.236).

### 3.19. Glycogen Phosphorylase, Muscle Form, PYGM

PYGM codes for the muscle-specific isoform of glycogen phosphorylase, an important enzyme in glycogenolysis, which facilitates the breakdown of glycogen into glucose-1-phosphate, thus playing a vital role in energy release during muscle contraction. The binding sites at residues 43, 76 and a stretch from 310 to 319, with confirmed affinity for adenosine monophosphate (AMP), indicate PYGM’s regulation through allosteric effects. AMP, as an allosteric activator, binds to these sites and signals PYGM to accelerate glycogen breakdown. Residues 109, 143 and 156 are noted for their involvement in the association of subunits, suggesting that these regions are critical for the oligomeric state of PYGM, which can exist as a homodimer or homotetramer [84]. The homodimer form is enzymatically active as phosphorylase B, whereas the homotetramer is phosphorylated to form the more active phosphorylase A.

**Figure 23:**
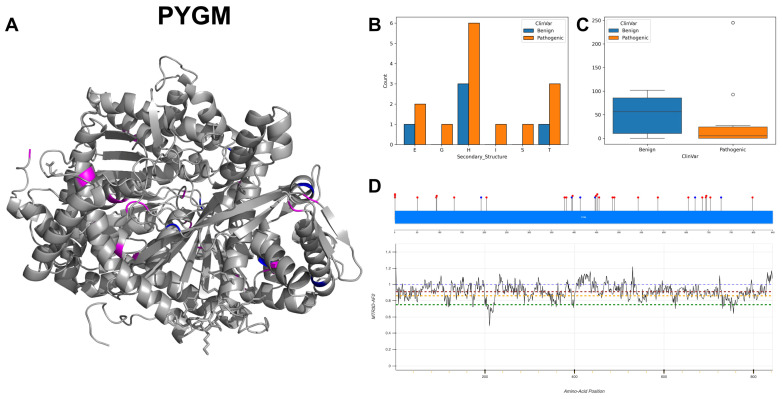
Structural analysis of the PYGM protein. (**A**) Localisation of benign variants in blue and pathogenic variants in magenta. (**B**) Annotated secondary structure variant mapping. (**C**) ACC distribution. (**D**) Assessment of ClinVar missense variants is presented in a lollipop plot, with benign variants in blue and pathogenic variants in red. The impact of these variants’ tolerance is evaluated through MTR3D-AF2, with green, yellow, red, and blue denoting the 5th, 25th, 50th, and neutrality (MTR = 1) percentiles, respectively.

PYGM exhibits a pattern of shifts in the GC content within nucleotide alterations, primarily comprising 11 instances of G to A changes and 5 occurrences of C to T mutations among the documented variants. The distribution of secondary structural elements in PYGM (Figure 23B), also shown in 3D structure visualisation (Figure 23A), predominantly consists of alpha-helices and extended strands, with a notable prevalence of helices. Solvent accessibility scoring visualisation indicates that benign variants of PYGM typically exhibit higher average accessibility to the solvent compared to pathogenic variants (Figure 23C). The majority of residues within PYGM are associated with MTR3D-AF2 scores ranging from 1 to 0.8 (Figure 23D), signifying a consistent minor level of missense intolerance throughout the gene. Variations in PYGM are documented in multiple studies, linking them with specific types of glycogen storage disease [85].

In assessing the molecular characteristics of PYGM pathogenic mutations (*n* = 24) relative to benign variants (*n* = 6), our investigation reveals that pathogenic mutations are not significantly closer to the protein core (*p*-value = 0.954) with a median ACC of 3 Å vs. 8.5 Å for benign variants. In terms of structural dynamics, the analysis indicates no significant difference in rigidity between pathogenic and benign variants, with median scaled deformation NMA values of 7.96 × 10^−5^ Å^2^ for pathogenic and 2.56 × 10^−4^ Å^2^ for benign variants (*p*-value = 0.35). Additionally, there is no significant difference in flexibility, as shown by median scaled fluctuation NMA values of 6.00 × 10^−5^ Å^2^ for pathogenic versus 1.62 × 10^−4^ Å^2^ for benign variants (*p*-value = 0.55). The evolutionary conservation analysis suggests that pathogenic variants have a median value of 0.798 compared to 1.383 for benign variants. However, this difference does not reach statistical significance (r4s *p*-value = 0.404), indicating that the variants are similarly conserved evolutionarily. Stability assessments using mCSM scores indicate that pathogenic variants tend to be less stable with a median score of −0.595 compared to −0.348 for benign variants. Yet, the difference in predicted stability between pathogenic and benign variants does not reach statistical significance (*p*-value = 0.455).

### 3.20. Sucrase-Isomaltase, Intestinal, SI

SI denotes sucrase-isomaltase, an enzyme that is integral to carbohydrate digestion and has dual functions, as indicated by its ability to act on different substrates: sucrose and isomaltose. The active sites at amino acid positions 505, 604, 1394, 1397 and 1500 are suitably adapted for the hydrolysis of the glycosidic bonds in sucrose and isomaltose, as evidenced by references to their nucleophilic and proton donor roles essential for isomaltase and sucrase activities, respectively. Binding sites at positions 264, 388, 588 and 662, linked with substrate recognition, suggest a complex interaction between SI and its substrates, facilitating effective catalysis. Sucrase and isomaltase activities are mediated by different subunits within the SI complex [86]. These subunits are non-covalently linked, which is consistent with SI’s role in the final stages of carbohydrate digestion, ready to act on disaccharides as they arrive in the intestinal lumen. The topological structure of SI, as a single pass type II membrane protein, is typical for enzymes anchored in the cell membrane. The transmembrane domain between amino acids 13 and 32 facilitates this anchorage, with the majority of the enzyme, from residues 33 to 1827, extending into the lumenal side.

**Figure 24:**
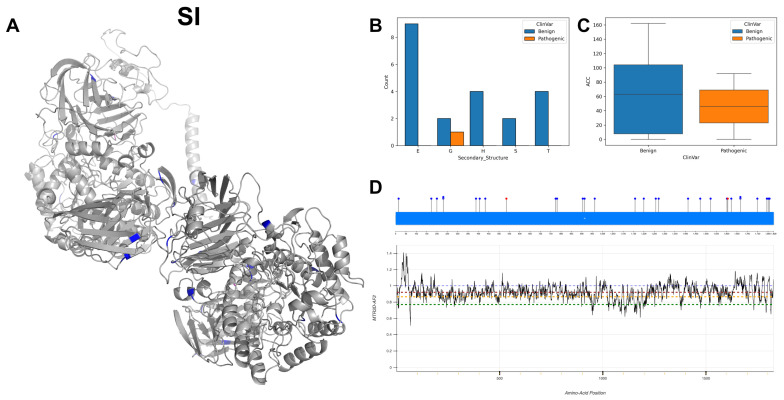
Structural analysis of the SI protein. (**A**) Localisation of benign variants in blue and pathogenic variants in magenta. (**B**) Annotated secondary structure variant mapping. (**C**) ACC distribution. (**D**) Assessment of ClinVar missense variants is presented in a lollipop plot, with benign variants in blue and pathogenic variants in red. The impact of these variants’ tolerance is evaluated through MTR3D-AF2, with green, yellow, red, and blue denoting the 5th, 25th, 50th, and neutrality (MTR = 1) percentiles, respectively.

SI shows slightly more transition than transversion nucleotide variants, primarily with G to A transition, followed by A to G and C to T transition. The secondary structure visualisation (Figure 24B) shows a significant preponderance of extended strands followed by alpha-helices. The solvent accessibility score (Figure 24C) shows that benign variants of SI tend to have a higher median ACC, indicative of a more solvent-exposed conformation, as opposed to pathogenic variants which exhibit a lower median ACC, suggesting a more buried localisation within SI’s three-dimensional structure. This is also visualised in the 3D structure (Figure 24A). The majority of residues across SI are associated with MTR3D-AF2 scores ranging between >1 and 0.8 (Figure 24D), suggesting that a minimal amount of missense intolerance is found throughout the gene. This aligns with the ratio of benign to pathogenic ClinVar variants found across the gene. Within SI, a total of 30 variants were documented, and the majority, specifically 28 variants, were classified as benign. This prevalence of benign alterations suggests a general trend towards non-pathogenic mutations. The documented findings imply a predominantly non-pathogenic nature with a rare occurrence of pathogenic alterations within the SI gene, specifically linked to sucrase-isomaltase deficiency [86].

In exploring the molecular characteristics of SI pathogenic mutations (*n* = 2) versus benign variants (*n* = 28), the studied results suggest that the median distances to active sites and binding sites are 33.23 Å and 32.22 Å, respectively. NMA shows a median deformation of 0.166 Å^2^ and a median fluctuation of 0.031 Å^2^ for regions housing benign variants. The r4s score median of 1.323 for benign variants indicates some degree of evolutionary conservation. Interestingly, a single pathogenic r4s score of 0.043 suggests a highly conserved residue, potentially indicating a critical site for protein function. The pathogenic mutations are associated with a less negative median mCSM score (−0.079) compared to benign variants (−0.99), suggesting that benign variants might have a greater predicted impact on protein function.

## 4. Conclusions

Despite the constraints in clinical data, both in terms of volume and the representation of benign versus pathogenic variants, our analysis reveals important structural aspects of glycoenzyme behaviour under variant conditions.

We found that glycoenzymes are particularly vulnerable to certain types of amino acid transitions, especially those occurring at the active sites critical for catalysis. Notably, transitions from “Negative” to “non-Negative” amino acids are particularly impactful. Additionally, our data indicate that glycine to cysteine mutations consistently result in pathogenic outcomes, highlighting the profound structural implications of these changes, particularly in terms of enzyme backbone and turn structures. The analysis of secondary structures reveals that pathogenic mutations predominantly manifest within beta bridges in contrast to benign variants, which display a high aggregate count; however, individual protein assessments suggest a lesser propensity for this trend and a broader diversity in mutational occurrences. Analysis performed using MTR3D-AF2 reveals the varying levels of intolerance that can be observed when comparing glycoenzymes to one another. Additionally, this analysis highlights regions of glycoenzymes that are likely to be vulnerable to disease-causing mutations, including those segments that lack sufficient variation data in the ClinVar database.

In examining the molecular consequences of disease-associated variants as opposed to those deemed benign, our findings suggest that disease-associated variants are often situated closer to the protein core. Additionally, these variants frequently lie near active and binding sites, which could impinge upon the protein’s interaction with substrates or other molecules. From a structural dynamic perspective, these variants are typically located in areas exhibiting higher rigidity or reduced flexibility. Conservation analysis indicates that disease-associated variants occur more commonly in evolutionarily preserved regions, highlighting their potential significance in critical protein functions that have been maintained throughout evolution. There is a general tendency towards decreased stability associated with the pathogenic variants, which also demonstrate a marked tendency to disrupt protein structure, which in turn, could affect function and stability, thereby contributing to the molecular pathology of various diseases.

Given the growing accessibility of sequencing data and advancements in structural prediction, our study underscores the necessity of understanding mutations from a structural viewpoint. This knowledge is crucial for comprehending disease mechanisms and developing effective therapeutic strategies. Our findings pave the way for future research in this domain, highlighting the need for a deeper exploration of glycoenzyme variants and their clinical implications. Our study shows the vital role of precise glycosylation progress prediction and also lays the groundwork for future research into therapeutic targets and the development of precision medicine strategies tailored to specific mutational profiles. These precision medicine strategies may include the design of molecules to stabilise the altered enzyme structure, enhance protein solubility or restore functionality, thereby offering targeted treatment options for glycosylation-related pathologies. Our study identifies potential therapeutic interventions for glycosylation-related disorders: devising small molecules to stabilise or offset the effects of mutated enzyme functions, developing molecular chaperones to aid in correct protein folding, utilising gene therapy to accurately correct mutations and engineering synthetic enzymes to bypass the effects of mutations. Moreover, our conservation and solvent accessibility analyses offer insights for targeting essential functional areas and designing medicinal compounds to safeguard destabilised protein structures. By laying the groundwork, we aim to facilitate the emergence of precision medicine interventions that are finely tuned to individual mutational profiles, with the potential to mitigate the effects of glycosylation-related diseases.

## Figures and Tables

**Table 1:** Summary of the top 20 human GT and GH variants with clinical significance from the ClinVar database.

Gene	UniProt ID	Protein Name	Family * (Fold)	Variant Counts	Number of Benign	Number of Pathogenic
GLA	P06280	Alpha-galactosidase A	GH27 (GH-D)	207	5	202
GAA	P10253	Lysosomal Alpha-glucosidase	GH31 (GH-D)	167	17	150
GLB1	P16278	Beta-galactosidase	GH35 (GH-A)	97	12	85
GALC	P54803	Galactocerebrosidase	GH59 (GH-A)	90	10	80
NAGLU	P54802	Alpha-N-acetylglucosaminidase	GH89	83	11	72
IDUA	P35475	Alpha-L-iduronidase	GH39 (GH-A)	75	14	61
GBA1	P04062	Lysosomal acid glucosylceramidase	GH30 (GH-A)	73	6	67
ALG13	Q9NP73	Putative bifunctional UDP-N-acetylglucosamine transferase and deubiquitinase ALG13	GT1 (GT-B)	56	50	6
EXT1	Q16394	Exostosin-1	GT47; GT64 (GT-B; GT-A)	49	15	34
HEXA	P06865	Beta-hexosaminidase subunit alpha	GH20 (GH-K)	45	4	41
GNE	Q9Y223	Bifunctional UDP-N-Acetylglucosamine 2-Epimerase/N-Acetylmannosamine Kinase		39	1	38
MUTYH	Q9UIF7	Adenine DNA glycosylase		38	12	26
MAN2B1	O00754	Lysosomal alpha-mannosidase	GH38	36	22	14
HEXB	P07686	Beta-hexosaminidase subunit beta	GH20 (GH-K)	33	13	20
GBE1	Q04446	1,4-alpha-glucan-branching enzyme	CBM48; GH13; (GH-H)	31	14	17
PIGA	P37287	Phosphatidylinositol N-acetylglucosaminyltransferase subunit A	GT4 (GT-B)	31	16	15
AGL	P35573	Glycogen debranching enzyme	GH13; GH133; (GH-H)	30	21	9
ALG1	Q9BT22	Chitobiosyldiphosphodolichol beta-mannosyltransferase	GT33 (GT-B)	30	14	16
PYGM	P11217	Glycogen phosphorylase, muscle form	GT35 (GT-B)	30	6	24
SI	P14410	Sucrase-isomaltase, intestinal	GH31 (GH-D)	30	28	2

* The information of family and mechanism is from the CAZy database.

## Data Availability

No new data were created or analyzed in this study. Data sharing is not applicable to this article.

## Supplementary Materials

The following supporting information can be downloaded at https://www.mdpi.com/article/10.3390/biom14040497/s1, Table S1: Statistics and Mann-Whitney Test Results of the Selected 20 Proteins.
