# Supplementary material for: Mutations in Glycosyltransferases and Glycosidases: Implications for Associated Diseases
Source: Biomolecules. 2024 Apr 19;14(4):497. doi: 10.3390/biom14040497 (PMC11048727; doi:10.3390/biom14040497)
Supplement: Supplementary file 1 [file biomolecules-14-00497-s001.zip › biomolecules-2795504-supplementary.pdf]

Supplementary Table S1. Statistics and Mann-Whitney Test Results of the Selected 20 Proteins

Gene Symbol	p-value of ACC	Median ACC (benign) (Å)	Median ACC (pathogenic) (Å)	p-value of average distance to active sites	Median average distance to active sites (benign) (Å)	Median average distance to active sites (pathogenic) (Å)	p-value of scaled deformation NMA	Median scaled deformation NMA (benign) (Å^2)	Median scaled deformation NMA (pathogenic) (Å^2)	p-value of scaled fluctuation NMA	Median scaled fluctuation NMA (benign) (Å^2)	Median scaled fluctuation NMA (pathogenic) (Å^2)
**SI**	1	0		1	33.2330874		1	0.16626622		1	0.03070661	
**GBA1**	1		7	1		24.1196872	1		0.04883343	1		0.01371153
**GLB1**	0.01405012	67	2.5	0.0969074	41.8651792	22.0194334	0.74710965	0.09583243	0.08088924	0.06415254	0.21747094	0.05739601
**NAGLU**	0.0028002	59.5	1	1			0.03375535	0.07206584	0.16742848	0.26487295	0.05966263	0.02404392
**MAN2B1**	0.91425199	0.5	1	0.036834	23.3790599	17.077541	0.20542404	0.00038162	0.00093061	0.35547624	0.00016475	0.00012428
**PYGM**	0.95423229	8.5	3	1			0.35042017	0.00025629	7.96E-05	0.5487395	0.00010622	6.00E-05
**EXT1**	0.01976634	35	6	0.00230668	22.9779392	48.8908816	0.04386739	0.06131663	0.15313596	0.00508127	0.18382015	0.05781039
**GBE1**	0.86970755	5.5	7	0.49225774	25.6974403	24.090489	0.63536464	0.19428173	0.15365389	0.87487512	0.07358066	0.08078677
**HEXB**	0.00515242	35	0	0.25364761	38.0654979	22.0809535	0.32981798	0.06635532	0.06459283	0.80213829	0.01257666	0.01226056
**HEXA**	0.02790553	62	22	0.12875992	27.9194211	23.2013117	0.16546543	0.25397702	0.08124286	0.84294197	0.04611765	0.07014505
**GAA**	2.36E-05	26.5	0	3.26E-05	37.5013096	19.0740277	0.49468364	0.1499508	0.12943835	0.00406719	0.06628381	0.0199118
**ALG1**	0.00283157	70	4.5	1			0.90252325	0.04207852	0.0365597	0.01011233	0.00434431	0.00145264
**GNE**	1		8	1		29.4781716	1		0.03612294	1		0.10245581
**GLA**	0.2526706	22	1	0.14529513	26.7747974	18.415886	0.0696695	0.47566919	0.22822138	0.15248676	0.09051871	0.06228933
**IDUA**	8.02E-05	19	3	0.07549415	31.893904	24.5852097	0.07822682	0.04731944	0.07056749	0.01714489	0.08519461	0.03307478
**GALC**	0.19080416	7.5	0	0.03280016	26.2699689	20.3383872	0.61626669	0.00478889	0.00333613	0.03820479	0.00070461	0.00036349
**MUTYH**	1		55	1		28.2525905	1		0.00198133	1		8.66E-05
**AGL**	1	39	4	0.67865468	40.968046	40.2261682	0.20646021	0.09857838	0.0488599	0.12920413	0.0310125	0.00750066
**PIGA**	0.024955	71	0	1			0.05613416	0.03834675	0.00123892	0.01554938	0.00293633	0.0007
**ALG13**	0.0505155	118	36.5	0.21512605	56.2035743	33.3303884	0.01344538	0.01834732	0.00218482	0.24201681	0.00319483	0.00088994

Supplementary Table S1. (Continue)

Gene Symbol	p-value of r4s	Median r4s (benign)	median_r4s (pathogenic)	p-value of mcsm	Median mcsm (benign)	Median mcsm (pathogenic)
**SI**	1	1.3231	0.0425	1	-0.99	-0.079
**GBA1**	0.01800997	1.5869	0.8573	0.03749412	-0.453	-0.996
**GLB1**	0.00449746	2.3393	0.6948	0.06488038	-0.531	-1.367
**NAGLU**	0.36561186	1.20285	0.765	0.0008403	-0.1955	-1.3765
**MAN2B1**	0.036834	1.07995	0.4347	0.91841029	-1.1965	-1.2225
**PYGM**	0.40396096	1.38345	0.7975	0.45461826	-0.3475	-0.5945
**EXT1**	0.00053873	1.6492	0.62415	0.00621024	-0.42	-0.817
**GBE1**	0.04987602	1.561	0.6297	0.04767292	-1.14	-0.1455
**HEXB**	0.00346185	1.8038	0.3797	0.28239726	-0.914	-1.195
**HEXA**	0.20193807	2.8168	1.37465	0.28079613	-1.208	-0.425
**GAA**	2.73E-06	2.33175	0.5158	0.00044443	-0.3955	-1.165
**ALG1**	0.00052024	2.7144	0.62415	0.23644472	-0.488	-0.619
**GNE**	1		0.43665	1		-1.742
**GLA**	0.00041587	3.5938	0.8409	0.68531321	-1.192	-1.067
**IDUA**	3.04E-05	2.5273	0.32515	0.3791618	-0.726	-0.887
**GALC**	0.00170792	1.4198	0.7962	0.98524972	-0.9665	-1.036
**MUTYH**	1		2.3243	1		-1.411
**AGL**	0.25441225	1.5301	0.9115	0.95304695	-0.9035	-0.908
**PIGA**	0.00042689	3.1956	0.6025	0.09904558	-0.399	-1.839
**ALG13**	0.16470588	1.6448	0.9939	0.02016807	-0.373	-1.566

[*] At least 2 data points from each group are required for the Mann-Whitney test. If the available data are not sufficient, the p-value is set to 1, indicating that there is no significance between the benign and pathogenic variant groups.
